# Indole-Based and Cyclopentenylindole-Based Analogues Containing Fluorine Group as Potential ^18^F-Labeled Positron Emission Tomography (PET) G-Protein Coupled Receptor 44 (GPR44) Tracers

**DOI:** 10.3390/ph16091203

**Published:** 2023-08-24

**Authors:** Runkai Yin, Kelly X. Huang, Lina A. Huang, Melinda Ji, Hanyi Zhao, Kathy Li, Anna Gao, Jiaqi Chen, Zhixuan Li, Tianxiong Liu, John E. Shively, Fouad Kandeel, Junfeng Li

**Affiliations:** 1 Department of Translational Research & Cellular Therapeutics, Arthur Riggs Diabetes and Metabolism Research Institute, Beckman Research Institute of the City of Hope, Duarte, CA 91010, USA; 2 Department of Immunology & Theranostics, Arthur Riggs Diabetes and Metabolism Research Institute, Beckman Research Institute of the City of Hope, Duarte, CA 91010, USA

**Keywords:** G-protein coupled receptor 44 (GPR44), cancer-related inflammation, inflammation-induced cancer, positron emission tomography (PET) imaging, fluorine-18, indole-based analogues, cyclopentenylindole-based analogues

## Abstract

Recently, growing evidence of the relationship between G-protein coupled receptor 44 (GPR44) and the inflammation-cancer system has garnered tremendous interest, while the exact role of GPR44 has not been fully elucidated. Currently, there is a strong and urgent need for the development of non-invasive in vivo GPR44 positron emission tomography (PET) radiotracers that can be used to aid the exploration of the relationship between inflammation and tumor biologic behavior. Accordingly, the choosing and radiolabeling of existing GPR44 antagonists containing a fluorine group could serve as a viable method to accelerate PET tracers development for in vivo imaging to this purpose. The present study aims to evaluate published (2000-present) indole-based and cyclopentenyl-indole-based analogues of the GPR44 antagonist to guide the development of fluorine-18 labeled PET tracers that can accurately detect inflammatory processes. The selected analogues contained a crucial fluorine nuclide and were characterized for various properties including binding affinity, selectivity, and pharmacokinetic and metabolic profile. Overall, 26 compounds with favorable to strong binding properties were identified. This review highlights the potential of GPR44 analogues for the development of PET tracers to study inflammation and cancer development and ultimately guide the development of targeted clinical therapies.

## 1. Introduction

### 1.1. Molecular Imaging of Inflammation in Cancer

Chronic inflammation is known as one trigger for the development of cancer, and it promotes all stages of tumorigenesis [1]. Several inflammatory molecules, signaling pathways, and cellular infiltrates play important roles in the biological behavior of tumors [2,3,4]. As a double-edged sword, inflammation responses may protect the body against foreign invaders in certain circumstances, but in other conditions, they may predispose the body to the onset of cancer when tumors escape such responses and utilize them to help their own replication [5]. Chronic inflammatory processes relate to all stages of tumor development as well as treatment [6], while oncogenic changes can generate an inflammatory microenvironment that promotes the development of tumors [7]. This vicious cycle is now well understood as including both cancer-related inflammation and inflammation-induced cancer. Direct cell-to-cell interaction as well as communication by inflammatory mediators such as cytokines or prostanoids underlies this interaction [8].

Novel mechanistic insights into the dynamic changes in the cellular physiology of inflammation that occurs in cancer is being pursued, with the goal of leveraging these results to reduce disease burden through the acceleration of tumor-specific therapeutic approaches and improved monitoring of therapeutic responses. Traditionally, clinical evaluations for cancer-associated inflammation rely mainly on immunological assays of biological fluids and tissue samples [9,10]. However, these diagnostic tools do not reveal dynamic tumor immune cellular physiology and inter-and intra-lesion heterogeneity in these interactions. Therefore, imaging technology plays an important role for its capability to monitor these tumor-inflammation interactions in vivo and in real-time in a non-invasive manner [11,12], which can be used not only for detection, but also for determining the inflammatory stage and precise location of premalignant lesions to aid in directing treatments. Furthermore, monitoring inflammation and tumor dynamics using such imaging technologies could provide the insight into cancer basic biology and accelerate drug development to combat inflammation [13].

Currently, there is a strong and urgent need for the development of non-invasive in vivo imaging techniques that can be used for these purposes. A vast range of molecular imaging strategies have been developed over the last decade, such as magnetic resonance imaging (MRI), positron emission tomography (PET), and single-photon emission computerized tomography (SPECT) [14]. Among these, PET uses tracer molecules with radioactively labeled nuclides so that they can be visualized in the body to determine molecular and functional processes, and is regarded as a highly sensitive and qualitative medical imaging technique [15]. Studies using PET alone [16,17,18] or in combination with other techniques, such as computerized tomography (CT) [19] and MRI [20,21], have shown the potential of this technique to be developed as a highly sensitive and qualitative medical in vivo imaging technique [19]. [^18^F]-fluorodeoxyglucose (FDG) has been established as a useful PET tracer in a number of studies. In 2009, Neumann et al. reported that PET coupled with [^18^F]-FDG was superior in revealing and thereby diagnosing inflammation in the large-vessel vasculitis, while conventional techniques such as MRI and computerized axial tomography (CAT) scans showed misleading results [22]. In 2015, Hess et al. reported a pilot study that showed PET coupled with [^18^F]-FDG could be used to evaluate the inflammatory process associated with venous thromboembolic disorders [23]. PET coupled with [^18^F]-FDG has also been used to study large vessel arterial inflammation [24] and fever of unknown origin in canines [19]. While the potential of PET in revealing inflammation in various diseases has been confirmed, the currently available markers, such as [^18^F]-FDG, have lacked specificity and resolution, and are insufficient for capturing low-grade inflammation processes [13]. Therefore, there is a need for specific PET biomarkers that can be used to track inflammation and tumor development with acceptable specificity and resolution [19,25].

### 1.2. G-Protein Coupled Receptor 44 (GPR44) in Cancer-Related Inflammation as Well as Inflammation-Induced Cancer

GPR44, also referred to as prostaglandin D2 receptor 2 (DP2) and chemoattractant receptor-homologous molecule expressed on T helper type 2 cells (CRTH2), was first isolated by Marchese et al. in 1999 [26,27]. It comprises seven transmembrane alpha helices, which has been recently mentioned in the literature [28,29]. As a lipid mediator, GPR44 is expressed in the gastrointestinal tract, brain, endocrine system, gall bladder, and muscle, but is also released in the immune system by immunoglobulin E (IgE)-activated mast cells, T-helper type 2 cells (Th2 cells), eosinophils, and dendritic cells [27,29,30,31,32]. In fact, GPR44 has been discovered to play a significant role in the process of inflammation, giving rise to inflammatory disorders of an array of organs [8]. It was reported that GPR44 underlaid the pathophysiology of asthma [8,30,31,33] such as inducing and amplifying the inflammation cascade [34], upregulating a series of inflammatory cytokines expression in Th2 cells [30], and activating eosinophils [30,31,35]. Currently, GPR44 has drawn increasing attention for the pathogenesis of cancer-related inflammation, inflammation-induced cancer, diabetes, inflammatory bowel disease, chronic kidney diseases, osteoarthritis, central nervous system diseases, and alopecia [8].

As an important mediator in the process of inflammation, GPR44 contributes to canceration in various systems of the human body according to previous research in lung cancer [36], gastric cancer [37], leukemia [38,39], osteosarcoma, colorectal cancer [40,41], breast cancer [42], multiple myeloma [43], and hepatocellular carcinoma [44]. For example, GPR44 activation can promote cardiomyocytes apoptosis through autocrine stimulation of reactive oxygen species and tumor necrosis factor α (TNF-α) production in a mitogen-activated protein kinase (MAPK) pathway-dependent way [45]. In apoptosis of human osteoclasts, GPR44 also plays a key role through extracellular signal-regulated kinase (ERK)1/2 and Akt signaling pathways [46]. Another study showed GPR44 expression and signaling in colorectal cancer tissue triggered proliferation, p-ERK1/2 and vascular endothelial growth factor (VEGF) expression and release, and increased metastatic activity, which could be used as a therapeutic target in colorectal cancer progression [40]. In a human study with a sample size of 80, data indicated that the mRNA expression level of the GPR44 was significantly increased in peripheral blood mononuclear cells (PBMCs) in patients with gastric cancer compared to healthy controls [47]. In a study on lung cancer using a mouse model, GPR44 expression was detected in vascular cells and growing tumor [48].

In 2001, binding assays revealed that the GPR44 agonist was pro-inflammatory mediator prostaglandin D_2_ (PGD_2_) [49,50]. PGD_2_ is a lipid molecule that has been implicated in asthma, allergic rhinitis, and other diseases involving type 2 inflammation. PGD_2_ binding to GPR44 causes an increase in intracellular levels of Ca^2+^, which inhibits cyclic adenosine monophosphate (cAMP) production and subsequently induces cell migration and activation [51]. In addition to GPR44 binding, PGD_2_ is also a ligand of D-prostanoid (DP_1_) and, when expressed at elevated levels, thromboxane receptor (TP). It was reported that the role of prostaglandin D_2_ (PGD_2_) in inflammation was medicated through its interaction with G-protein coupled receptors. When signaling through GPR44 receptor, PGD_2_ became pro-inflammatory in chronic helminth infections [52] and allergic reactions [53,54].

While the exact role of GPR44 in the inflammation-cancer system has not been fully elucidated, these studies indicated the tumorigenic properties of GPR44. Therefore, GPR44 is a promising target to aid the further exploration of the relationship between inflammation and tumor biologic behavior.

### 1.3. GPR44 and Fluorine-18 PET Tracers

**Figure 1:**
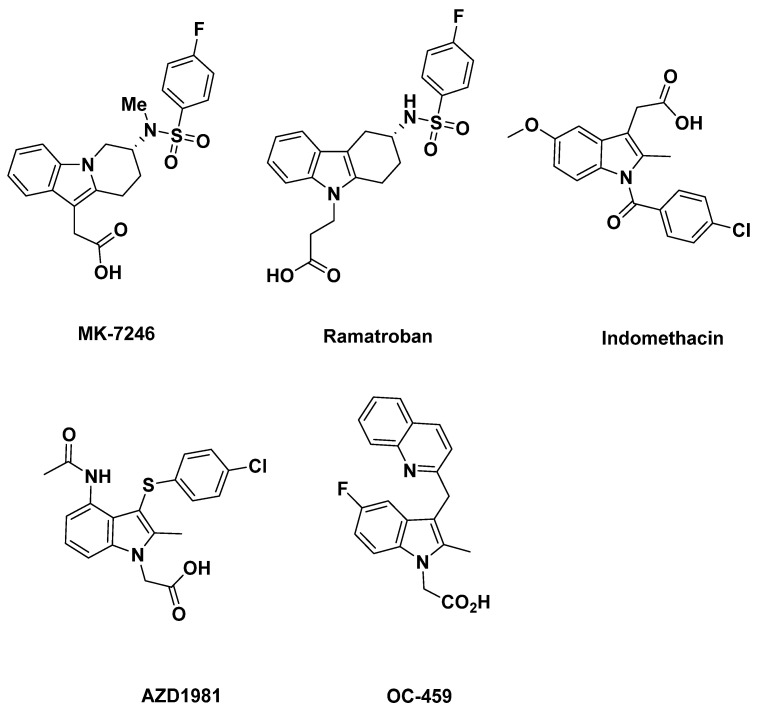
Molecular structures of compounds MK-7246, Ramatroban, Indomethacin, AZD1981, and OC-459.

Currently, carbon-11 has been mostly used to radiolabel GPR44 antagonists for PET imaging. For example, AZ12204657 [55] (IC_50_ = 2.5 nM and Kd = 2.9 µg) and MK-7246 [56] (Figure 1), molecules with high affinities GPR44, are carbon-11 labeled antagonists. Although using ^11^C-labeling GPR44 PET tracers in animals has been shown to be effective in research, there are many limitations with using ^11^C-labeling for PET tracers. One of the main drawbacks of using carbon-11 is its half-life of only 20 min in addition to a viability of only 2 h. Because of its short half-life and viability time, mass production and use of a carbon-11 labeled tracer is highly inefficient. Therefore, using carbon-11 radiolabels faces a major limitation in practical use, hindering the extent of their applicability. Additionally, as the clinical use of PET tracers needs to be completed within three half-lives and the requirement that carbon-11 has to be artificially produced by an on-site cyclotron, administrations of these tracers to multiple patients, delivery to remote sites, and use in research are severely hindered.

^18^F-labeling is preferable over ^11^C-labeling due to its significantly greater half-life of 110 min, which allows for up to 10 h of PET imaging. The longer half-life and viability thus enable its mass production and distribution [57]. Using fluorine-18 is also beneficial given its positron emission (β+ decay) of 97%, favorable van der Waals radius (1.47 Å), low maximum positron energy (0.635 MeV), and high electronegativity. In addition, its high-energy bond with carbon allows increased tracer stability at higher temperatures and better oxidation resistance. The advantages of ^18^F-labeling compared to labeling with other radionuclide include better in vivo biological properties and biological behavior [58,59].

In our previous report, we summarized ramatroban-based GPR44 ligands containing a fluorine group and assessed their potential as targets for the development of novel ^18^F-labeled PET tracers [60]. The present paper aims to continue to summarize current indole-based potential GPR44 analogues and cyclopentenylindole-based potential GPR44 analogues containing a fluorine group and evaluate their potential as treatment targets, to provide a more complete map of current GPR44 analogues containing a fluorine group as options for cancer-related inflammation PET tracer development.

## 2. Indole-Based Potential GPR44 Analogues

In this section, we evaluate a series of known analogues containing an isolated indole group not fused with other rings, with the exception of those already discussed as ramatroban (Figure 1) and ramatroban-based analogues [60]. These compounds could be considered as analogues of the well-characterized GPR44 antagonist indomethacin (Figure 1), a nonsteroidal anti-inflammatory drug (NSAID) commonly used to reduce fever, pain, stiffness, and swelling from inflammation [61]. Indomethacin is not described here due to its lack of a fluorine atom that may allow for ^18^F radiolabeling.

For substantially the same reasons, another indole-based GPR44 antagonist developed for treating respiratory diseases including asthma, AZD1981, is not discussed in more detail here due to the lack of a fluorine atom [62].

### 2.1. Ono Pharmaceutical Co Analogues

#### 2.1.1. Ono Pharmaceutical Co Analogues in 2004–2005

**Figure 2:**
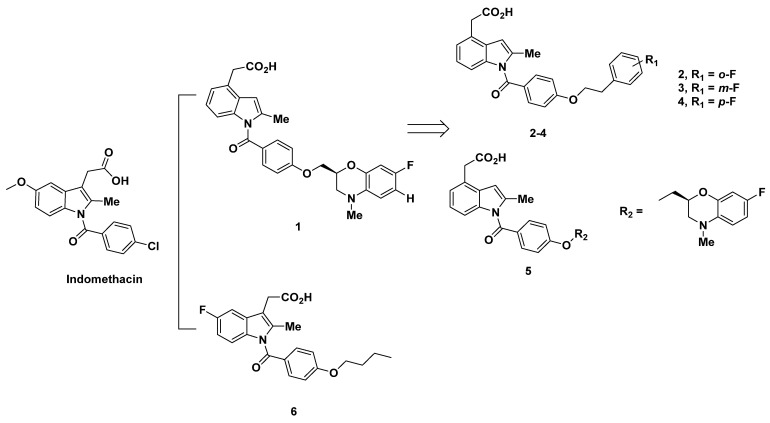
Molecular structures of compounds **1**–**6**.

In light of the correlation of PGD_2_ with allergic diseases, Torisu et al. at Ono Pharmaceutical Co developed a series of human prostaglandin D_2_ receptor antagonists in 2004 [63]. Among the antagonists, compounds **1**–**4** were created with a 4-position acetic acid residue from *N*-benzoyl-2-methylindole and change in the *p*-alkoxy moiety, corresponding to the lipophilic moiety of PGD_2_ receptors (Figure 2).

Table 1 showed the greatest binding potential to DP, with a K_i_ of 5.3 nM and IC_50_ of 0.81 nM. The *o*-fluoro derivative **2** exhibited the next best affinity and selectivity for DP at a moderate K_i_ of 46 nM and an IC_50_ of 150 nM. *m*-Fluoro compound **3** and *p*-fluoro compound **4** both exhibited a similar subpar K_i_ and IC_50_, each at a value greater than 130 nM (hDP, Table 1).

Although compounds **1**–**4** all exhibited the selectivity for DP, their bindings to targets DP_1_ and GPR44 were not discussed in this research. Due to the lack of evidence of selectivity for GPR44, it is uncertain if these compounds can be further developed as PET tracers for GPR44, as the compounds may have the risk of off-target binding to DP_1_.

In pursuit of an optimal DP_1_ antagonist, Torisu et al. further formulated a series of *N*-benzoyl-2-methylindole-4-acetic acids from a *p*-phenyloxy-ethyloxy derivative in 2004 [64]. The compound with good binding to DP_1_ was identified as compound **5**, evaluated through [^3^H]PGD_2_ and cAMP inhibition binding assays. The 4*N*-methylbenzo-morpholinyl-2-methyloxy analogue **5** demonstrated weak binding to EP, with K_i_ values for EP_1–4_ all above 1.7 µM. The binding to DP_1_, meanwhile, was better with a K_i_ of 5.3 nM and IC_50_ of 0.81 nM. In vivo testing of compound **5** for inhibition of PGD_2_-induced vascular permeability in guinea pig conjunctiva showed that a 0.3 mg/kg oral dose provoked 60% inhibition [64].

In 2005, Torisu et al. continued the search for a selective DP_1_ antagonist modified from indomethacin analogues and specifically created from 4-substituted phenylhydrazines. The K_i_ of compound **6** for DP_1_, measured through an [^3^H]PGD_2_ binding assay, was subpar at 110 nM. Correspondingly, the IC_50_ for DP_1_ was 1600 nM (mDP) [65]. However, selectivity for DP_1_ was high in that the K_i_ values for binding to EP_1–4_ were all more than 10 µM, with the exception of 2.8 µM for EP_2_. Of note, all the prostanoid receptors in this study were derived from mice [65].

#### 2.1.2. Recommendations

Although compounds **1**–**6** all exhibited the selectivity for DP, there is unfortunately no data on the affinity and selectivity to GPR44. These compounds, therefore, need further characterization for development as GPR44 PET tracers.

#### 2.1.3. Ono Pharmaceutical Co Analogues in 2011

In 2011, Iwahashi et al. from Ono Pharmaceutical Co. synthesized novel DP_1_ antagonists based on indomethacin [66]. The K_i_ and IC_50_ values of the compounds were determined through [^3^H]PGD_2_ and cAMP inhibition assays, respectively (Table 2). Of note is that the values were elucidated through binding to a mouse prostanoid receptor.

The 5-fluoro-included 2-methyl indole-3-acetic acid **7** exhibited a particularly high affinity for DP_1_, with a K_i_ of 5.7 nM and IC_50_ of 1.8 nM. Compound **7** was also shown to be specific for DP_1_, with a K_i_ above 250 nM for binding to other prostanoids (EP_1–4_, FP, TP, and IP).

**Figure 3:**
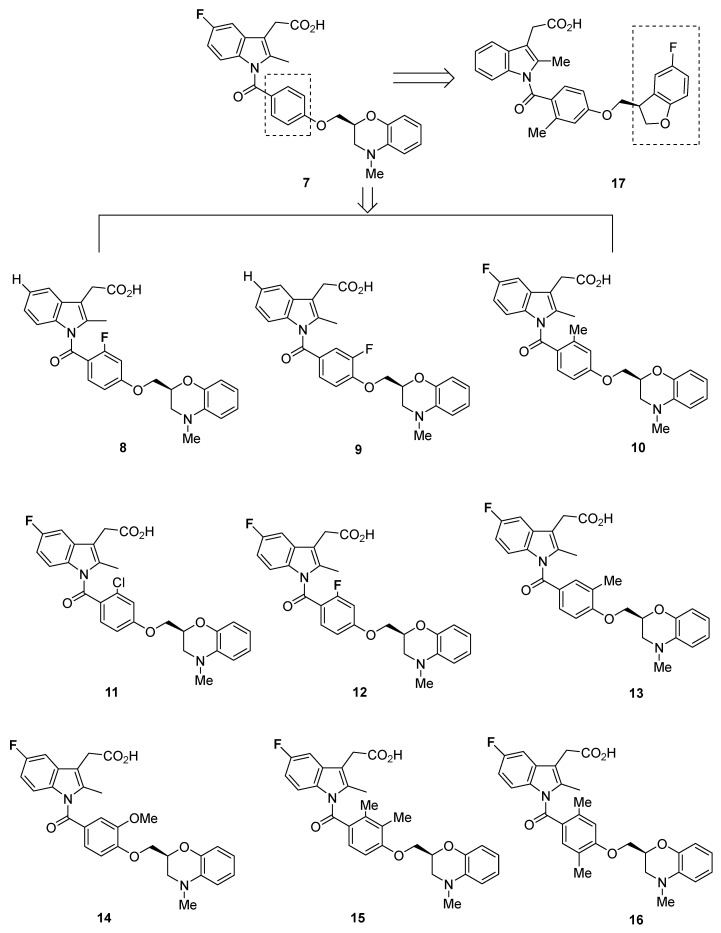
Molecular structures of compounds **7**–**17**. The dashed box (compound **7**) highlights the differences between compound **7** and compounds **8–16**. The dashed box (compound **17**) highlights the substitution of a benzofuran-3-yl moiety.

Further modifications of the indole and phenyl groups yielded compounds **8**–**16** (Figure 3). With the exception of **9** (K_i_ = 240 nM), **13** (K_i_ = 59 nM and IC_50_ = 42 nM), **14** (IC_50_ = 68 nM), **15** (IC_50_ = 32 nM), and **16** (K_i_ = 14 nM and IC_50_ = 2.8 nM), all compounds had K_i_ and IC_50_ values below 6 nM. Although the binding affinity for DP_1_ appeared to be high in these compounds, the selectivity was not as pronounced as would be optimal, with the K_i_ for human IP at values ranging from 93 to 320 nM (with the exception of **14** with its K_i_ of 1.5 µM).

Substitution of a benzofuran-3-yl moiety led to compound **17** (Figure 3), which had similarly low K_i_ and IC_50_ values (5.4 and 1.8 nM, respectively) but suboptimal selectivity (IP K_i_ = 520 nM) [66].

#### 2.1.4. Recommendations

The compounds developed here were only tested as DP_1_ antagonists, and were not characterized for binding affinity to GPR44. However, on the basis of the high affinity to DP_1_, these compounds are not promising for use as GPR44 PET tracers.

### 2.2. Oxagen Analogues

#### 2.2.1. Oxagen Analogues in 2005 and 2007

**Figure 4:**
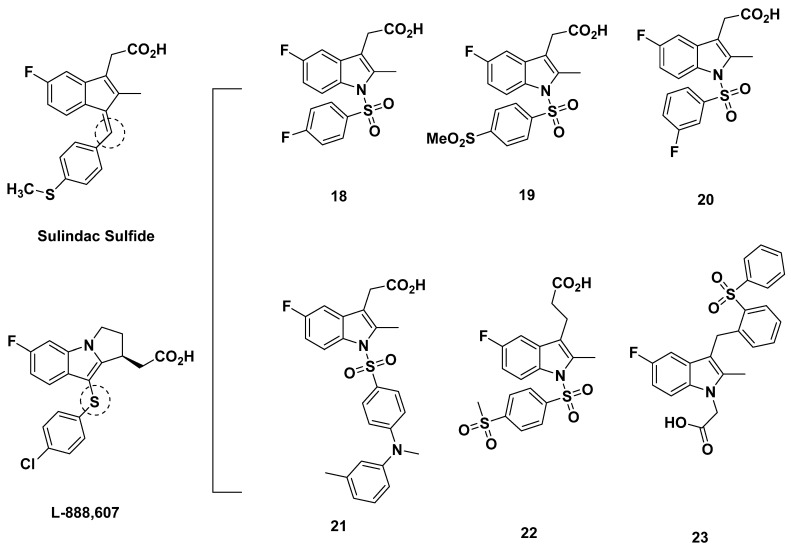
Molecular structures of compounds sulindac sulfide, L-888,607, and **18**–**23**. The dashed circles highlight the differences in the linker.

In 2005, Armer et al. from Oxagen aimed to discover a selective antagonist of GPR44 based on a common indole acetic acid structure [67]. The molecules were designed around a (5-fluoro-2-methyl-1H-indolyl3-yl) acetic acid core due to the promising binding affinities of sulindac sulfide and L-888,607 (Figure 4) [68,69], compounds which shared this core, and the optimal structure-activity relationship (SAR) of compound libraries sharing the core. The addition of an *N*-sulfonyl group was further characterized to increase affinity, and the novel compounds **18**–**20** (Figure 4) were created as such.

Compounds **18** and **20** both exhibited selectivity, with binding to DP_1_ low at K_i_ values greater than 6 µM. However, the binding affinity to GPR44, though better, was found to be also low at 71 and 225 nM, respectively. Reflecting the low K_i_ values, the IC_50_ for inhibition of PGD_2_-induced Ca^2+^ flux was above 150 nM for both compounds [67].

Compound **19** showed better affinity with an improved K_i_ of 68 nM, a Ca^2+^ IC_50_ of 19 nM, an eosinophil shape change assay IC_50_ of 74 nM, and a Th2 cell chemotaxis IC_50_ of 67 nM. In rat and human microsomes, compound **19** did not exhibit concerning levels of metabolism, with no cytochrome P450 (CYP450) isoforms inhibited or induced at a dose of 1 µM. Compound **19** additionally did not exhibit cytotoxicity in the HepG2 hepatocarcinoma cell line, nor influence in a human ether-a-go-go-related (hERG) channel assay, which marks potential inducement of the heart condition acquired long QT syndrome (LQTS). In rats, intravenous and oral administration revealed promising pharmacokinetic properties. Intravenous administration of 1 mg/kg yielded an acceptable C_max_ of 1801 ng/mL, an area under the curve (AUC) of 619 ng/(mL·h), a clearance of 40 (mL/min)/kg, and a half-life of 2.2 h. The oral dose of 10 mg/kg yielded a five-times lower C_max_ at 1.8 h, four-times higher AUC, a half-life of 5.5 h, an acceptable bioavailability of 56%, and unidentified clearance. The discrepancy may be due to the differing ways of administration, as well as the ten-times higher oral dose [67].

Oxagen also disclosed in a patent publication the 1-acetic-acid derivative (compound **21**) with a sulphonyl spacer between the indole and aromatic groups. However, this compound exhibited a low affinity and selectivity for GPR44, with its K_i_ of 222 nM for GPR44 and a K_i_ of 86 nM for DP_1_, indicating potential off-target binding [31].

#### 2.2.2. Recommendations

Due to the low affinity for GPR44, shown by the high K_i_ and IC_50_ binding values, compounds **18**–**21** may not be optimal compounds to serve as efficacious GPR44 PET ligands.

#### 2.2.3. Oxagen Analogues in 2010

In a 2010 review [70], Norman described an indole-1-sulfonyl-3-acetic acid developed by Oxagen (compound **22**) in Figure 4 that was similar to compound **19** created by Armer et al., save for the replacement of the acetic group attached to the aromatic ring by a propanoic group [67]. The IC_50_ value of the compound was subpar at 68 nM, but the selectivity for GPR44, as opposed to other prostanoid receptors, was adequate.

Norman additionally reviewed compound **23**, disclosed by Oxagen in a patent filing, whose properties were not discussed [70,71].

#### 2.2.4. Recommendations

As such, due to the paucity of information regarding compounds **22**–**23**, these compounds need further characterization before development for use as PET tracers. However, compound **22** could be reasonably disregarded due to the subpar binding affinity.

#### 2.2.5. OC-459 (OC000459/Timapiprant)

In 2012, Pettipher et al. from Oxagen characterized a 3-quinolinylmethylindol-1-yl-acetic acid, in which the indole scaffold was reversed [72]. This compound, known as OC-459, OC000459, and Timapiprant, is a selective GPR44 antagonist (Figure 1). Of note, the structure included in this review has not been confirmed by Oxagen, but was characterized as OC-459 by Amira. In addition, although Pettipher et al. were the first to publish a non-clinical study of OC-459, it appears that the unpublished development of OC-459 had already been undertaken by other parties [73,74,75,76,77,78].

In a [^3^H]PGD_2_ competition binding assay in Chinese hamster ovary (CHO) cells, OC-459 showed a high binding affinity for GPR44 at a K_i_ of 13 nM. The K_i_ value on the native receptor in human Th2 lymphocytes was similarly low at 4 nM. The IC_50_ value of Ca^2+^ inhibition and Th2 lymphocyte chemotaxis was consistent with the K_i_ values at 28 nM. OC-459 was also shown to inhibit IL-13 production by Th2 cells at an IC_50_ of 19 nM and inhibit PGD_2_-induced Th2 cell survival at an IC_50_ of 35 nM. For eosinophil shape change, OC-459 inhibited the response at an IC_50_ of 11 nM.

The [^3^H]PGD_2_ displacement in rat GPR44 was comparable at 3 nM, indicating that rats may be used as an in vivo model without major concerns. Selectivity was also promising, as the K_i_ or IC_50_ values for OC-459 binding to DP_1_, EP_1–4_, FP, IP, and TP were all above 10 µM. A concentration of 10 µM OC-459 also exerted no effects on 69 receptors, ion channels, transporters, and 17 enzymes. These enzymes included the cyclooxygenases cyclooxygenase 1 (COX1) and cyclooxygenase 1 (COX2), in recombinant form, and the native COX1, as expressed in platelets.

OC-459 was also characterized for plasma protein binding and found to bind strongly at 99.1% and 99.8% in human and rat plasma, respectively. The pharmacokinetic profile of OC-459 was elucidated in male Sprague-Dawley rats. At a dose of 2 mg/kg intravenously, OC-459 had a C_max_ of 5660.4 ng/mK at 0.08 h, a half-life of 1.3 h, a clearance of 8.4 mL/kg/min, an AUC_24h_ of 4486.5 ng/mL·h, a volume of distribution of 0.5 L/kg, and an undiscerned bioavailability. At the same concentration dosed orally, the C_max_ was 1543 ng/mL at 1.3 h, the half-life was 2.9 h, the clearance was 6.4 mL/kg/min, the AUC_24h_ was 5262.5 ng/mL·h, the volume of distribution was not found, and the bioavailability was high at 111%. Of the values elucidated, all seemed characteristic of a well-absorbed and promising compound.

The pharmacologic profile of OC-459 was briefly delved into, and it was characterized as a competitive antagonist. OC-459 also appeared to dissociate at a quick rate from GPR44 with a half-life of 0.41 min [79].

The inhibitory effects of OC-459 were further elucidated in rats orally administered OC-459, which reduced 13,14-dihydro-15-keto-PGD2 (DK-PGD_2_)-induced eosinophil count. Similarly, eosinophil numbers were reduced following OC-459 oral administration in guinea pigs.

As the first GPR44 antagonist found to act clinically on allergic disease, OC-459 was initially characterized in clinical studies by Barnes et al. in 2012 [80]. This double-blind, parallel group trial included 65 subjects with moderate persistent asthma who were given 200 mg of OC-459 twice daily. The compound ended up gradually increasing its forced expiratory volume in 1 s (FEV1). In addition, OC-459 reduced circulating IgE and night-time symptoms. The plasma concentration of OC-459 was detected as 600 ng/mL, indicating that the GPR44 receptors had been successfully blocked. Importantly, OC-459 was shown to have a tolerability comparable to that of the placebo. In essence, the clinical study confirmed the promising properties of OC-459 from in vivo preclinical models, and established OC-459 for treatment of disease [81,82].

In 2012, a single center, randomized, double-blind, placebo-controlled, two-way crossover study was published [80]. It indicated that OC-459 could reduce symptoms of allergic rhinoconjunctivitis, at least in those allergic to grass pollen. Again, the compound was well-tolerated and acted more quickly than in the previous study, even though the dose was still 200 mg twice daily. An unexpected benefit to OC-459 administration was persistent inhibition, lasting up to 4 weeks. The response may be due to the apoptosis and clearance of Th2 lymphocytes induced by GPR44 inhibition, as OC-459 has a half-life of less than a day [83].

Further clinical studies characterized OC-459 as an inhibitor of eosinophils in corticosteroid-dependent or corticosteroid-refractory eosinophilic esophagitis patients and an inhibitor of the late asthmatic response, sputum eosinophil number and eosinophil shape change from steroid-naive asthmatic patients [84]. In mice, OC-459 also reduced inflammation and its corresponding markers, eosinophil migration, and symptoms of 2,4,6-trinitrobenzenesulfonic acid (TNBS)-induced colitis [85].

However, in 2010, progress on OC-459 decreased following licensing of rights to Eleventa and Atopix, reportedly due to financial concerns. Later, another study published in 2021 found that OC-459 treatment had little impact on the clinicopathological changes induced by rhinovirus RV-A16 infection in patients treated with inhaled corticosteroids, though the same study also concluded that OC-459 was safe and well-tolerated [76].

#### 2.2.6. Recommendations

Although the K_i_ of OC-459 is not as low as other promising compounds, the extensive characterization of comparable IC_50_ values and good safety profile make it attractive for further development as a GPR44 PET tracer.

### 2.3. AstraZeneca Analogues

**Figure 5:**
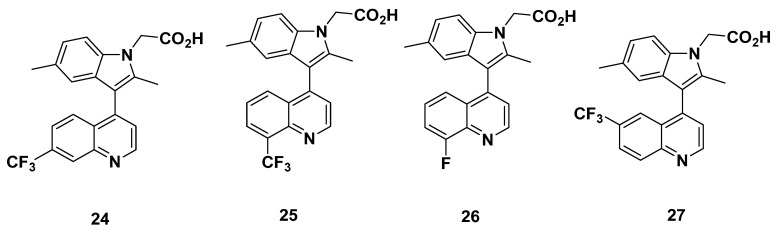
Molecular structures of compounds **24–27**.

Birkinshaw et al. from AstraZeneca derived a series of indomethacin-based compounds in 2006 (Figure 5) [86]. In [^3^H]PGD_2_ displacement assays, the IC_50_ of most of the compounds (**24**–**26**) were suitably low at 5.4, 6.0, and 7.1 nM, respectively; the IC_50_ of compound **27**, meanwhile, was high at 68 nM, indicating the low binding affinity of compound **27**. Apart from in vivo potency studies, Birkinshaw et al. calculated the whole blood potency of the compounds, a marker of plasma protein binding. Except for the unmeasured compound **27**, the whole blood potency was high at values ranging about 790 nM. This indicates the possibly low activity of the compound in blood. As could be predicted, the plasma protein binding was high at a value greater than 99.1 nM. The lipophilicity of all compounds, however, was acceptable at values from 0.5 to 1.8, measured as log D_7.4_. In vitro assessment of clearance yielded low values below 2.6 µL/min/10^6^ cells in human hepatocytes (with the exception of compound **27,** which again, could not be identified) but extremely divergent values in rat hepatocytes. The values ranged from <1 µL/min/10^6^ cells for compound **26**, 6.5 µL/min/10^6^ cells for compound **25**, and above 16 µL/min/10^6^ cells for compounds **24** and **27**. The discrepancy in values between human and rat cells may point to species-dependent clearance, but the consistently low clearance in human hepatocytes is promising, as it indicates an acceptable compound metabolism.

#### Recommendations

Due to the high inhibitory potential of compounds **24**–**26**, all these compounds may be assessed as potential GPR44 PET tracers. The high whole blood potency and discrepancy in rat and human clearance measurements may be taken into consideration in the development, but should not preclude compounds **24**–**26** from consideration [86].

### 2.4. AstraZeneca Analogues

**Figure 6:**
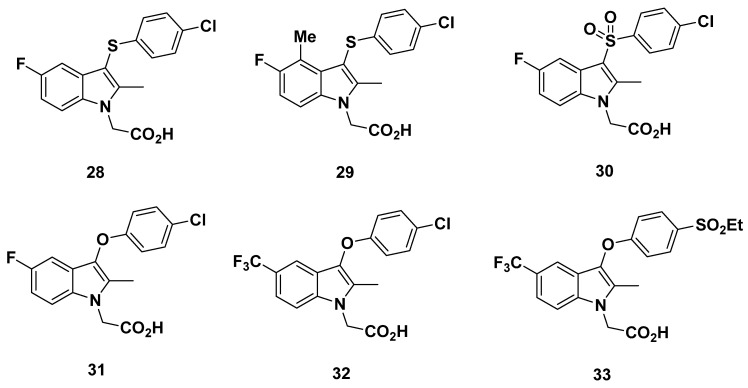
Molecular structures of compounds **28**–**33**.

In 2011, Luker et al. from AstraZeneca developed a series of novel GPR44 antagonists based on a 3-thioaryl indole found through screening AstraZeneca compounds similar to indomethacin (Figure 6) [87].

Examination on substitutions led to compounds **28**–**29**, both of which exhibited a suitable affinity for GPR44 at IC_50_ values of 1.6 and 1.3 nM, respectively (as measured through a [^3^H]PGD_2_ assay). To determine specificity, binding to human aldose reductase (ALD2), which had been shown to be inhibited by indomethacin, and related aldehyde reductase (ALD1) were evaluated. ALD2 values were adequately large at >7.9 µM and 3.5 µM, respectively. ALD1 values were slightly lower at 3.1 µM and 2.2 µM, respectively. The clearance from rat hepatocytes (19 and 17 µL/min/10^6^ cells, respectively) and human liver microsomes (<3 µL/min/mg for both compounds **28**–**29**) was also assessed. These low clearance values are preferable properties.

Incidentally, compound **28** has been disclosed as a compound developed by Pettipher et al. from Oxagen [88]. The K_i_ of the compound was reportedly 5 nM [89].

In contrast to compounds **28**–**29**, sulfone and oxygen-linked compounds **30**–**33** had a less optimal affinity for GPR44, with IC_50_ values of 7.1, 5.0, 11, and 3.5 nM, respectively. For all compounds save for compound **33**, the aldose reductase (ALR2) and aldehyde reductase (ALR1) selectivities were unable to be determined. Compound **33** showed an optimal binding with an IC_50_ value greater than 10 µM for ALR2 but only at 3.9 µM for ALR2. This selectivity was interesting in that other compounds in the study with the same aryloxy side chains as compound **33** showed an even lower IC_50_ for ALR1 for reasons undetermined [87].

#### Recommendations

The high affinity of compounds **28**–**29**, **31** and **33,** with an IC_50_ of no more than 5 nM, makes them promising for development into GPR44 PET tracers. Compound **32**, with its IC_50_ of 11 nM, may also be considered. However, off-target binding to ALR2 and ALR1 must be taken into account, and further characterization of binding affinity to other prostanoid receptors, as well as in vivo pharmacokinetics, would be recommended.

### 2.5. Pfizer Analogues

#### 2.5.1. Development of Analogues

**Figure 7:**
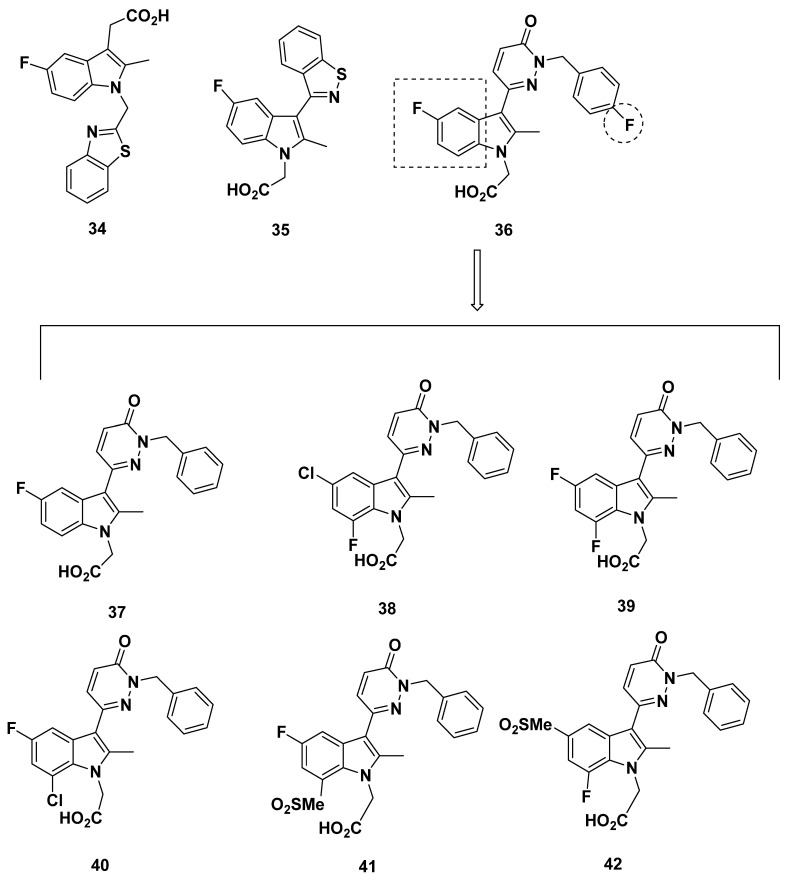
Molecular structures of compounds **34**–**42**. The dashed box and dashed circle highlight the differences between compound **36** and compounds **37–42**.

In a patent published in 2002, Pfizer disclosed compound **34** (Figure 7), which was reported to have 40-fold selectivity for GPR44 over DP_1_ [90]. Similarly, compound **35** (Figure 7) was reported to have a GPR44 pIC_50_ of 8.15.

In 2012, Kaila et al. from Pfizer developed a series of compounds based on diazine indole acetic acids [91]. Synthesized to inhibit GPR44 to reduce symptoms of asthma, the compounds were each subjected to a cAMP hGPR44 fluorescence resonance energy transfer (FRET) assay in vitro, and those with promising results were further subjected to pharmacokinetic characterization.

The substitution of the 5-position of the indole and 3-position of the diazine in compound **36** (Figure 7) resulted in a moderate affinity for GPR44 at an IC_50_ of 20 nM. The lipophilicity at a calculated partition coefficient (clogP) of 3.5 was also promising, as well as an unconcerning microsomal stability, an indicator of metabolism and clearance rate, at greater than 30 min. As might be inferred by the microsomal stability value, the clearance was low at 6.9 mL/min/kg in mice. However, the bioavailability of 11% precluded further studies on compound **36**, as well as a 0.3 × 10^−6^ cm/s in a Caco-2 cell assay, which measured intestinal absorption. The low value, reflecting a low level of absorption, may be expected from the lipophilicity.

The modification of compound **36** through the addition of hydrophobic groups resulted in the development of compound **37** (Figure 7), which exhibited a moderate affinity for GPR44 at an IC_50_ of 23 nM. The lipophilicity was again on the higher end of the acceptable level, with a clogP of 3.33. The ionization of the compound, measured through pK_a_, was 4.02, indicating that it contained an ionizable group. The Caco-2 cell assay found that compound **37** exhibited permeability of 0.5 × 10^−6^ cm/s, albeit at a reduced pH of 6.5, the permeability was reportedly reduced due to the carboxylic acid moiety of the compounds.

Substitutions at the 7-position of the A ring of the indole core reduced affinity in compounds **38**–**42** (Figure 7). In these compounds, the IC_50_ was not ideal, with values above 76 nM and even rising to greater than 1000 nM. Thus, although the lipophilicity stayed relatively constant at a clogP ranging from around 1 to 4 and the pK_a_ was around a value of 3.8, the low affinity of these compounds to GPR44 disqualified them from further characterization.

**Figure 8:**
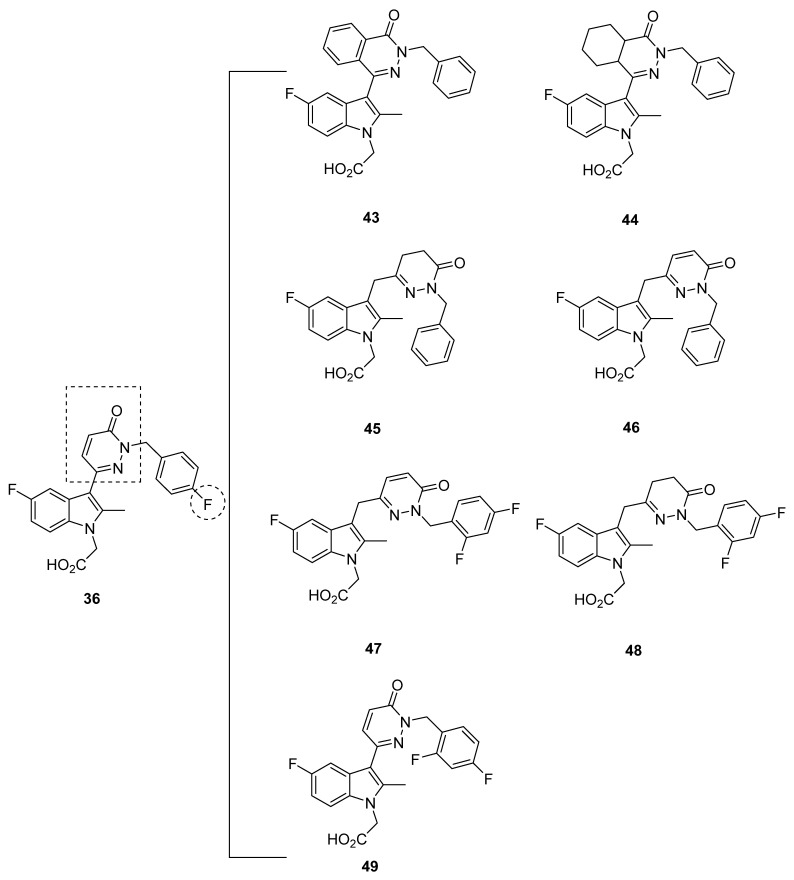
Molecular structures of compounds **36**, **43**–**49**. The dashed box and dashed circle highlight the differences between compound **36** and compounds **43–49**.

Reduction of the diazine ring was explored in hopes of increasing membrane permeability, resulting in several compounds in which affinity was compromised (compounds **43**–**44**) while others were promising (Figure 8). The IC_50_ of compounds **43**–**44**, as well as compound **45**, were all above 11 nM. Unfortunately, a discrepancy in the data for compound **45** was exhibited, in which the 16 nM IC_50_ of compound **45** was later labeled as 1.5 nM instead. Due to further studies with the eosinophil shape change assay described later, however, we decided to consider compound **45** as promising.

On the other hand, the IC_50_ values for compounds **46**–**48** were 9, 3.8, and 4 nM, respectively. A compound described briefly, compound **49**, also had a moderate IC_50_ of 10 nM. The solubility of compounds **47**–**48** in aqueous solution was further determined at a pH of 6.6, yielding 0.318 and 0.954 mg/mL; these values may inform development of the compounds, once made into tracers, about their dissolvability in solution.

**Figure 9:**
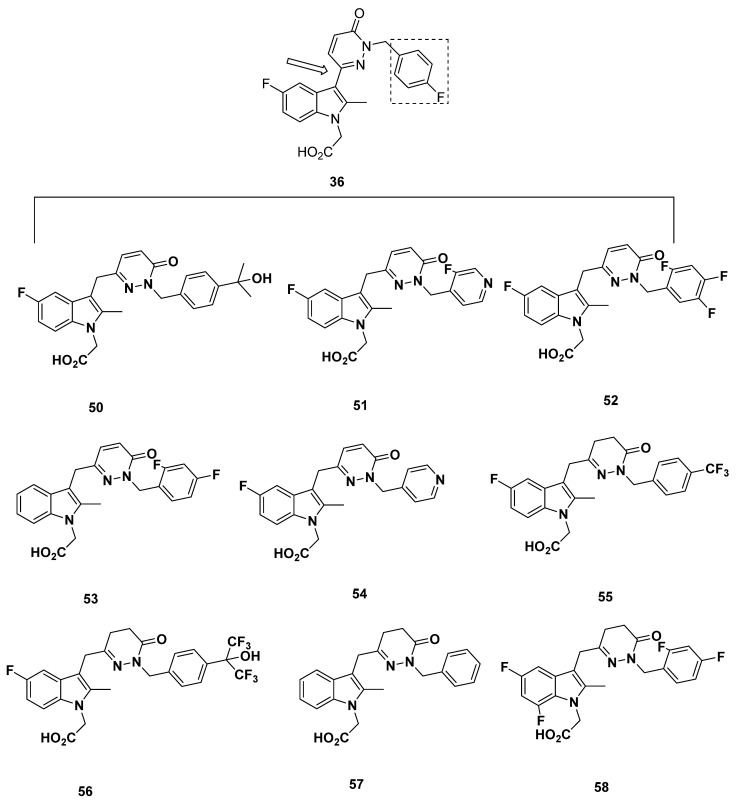
Molecular structures of compounds **36**, **50**–**58**. The dashed box highlights the differences between compound **36** and compounds **50**–**58**. The arrow indicates the change in the linker.

Additional compounds **50**–**58** with a pyridazine linker were developed (Figure 9). The IC_50_ of compounds **53**–**55** and **57**–**58** in both the FRET assay and the eosinophil shape change assay were all above 10 nM, with the lowest being 12 nM and the highest being 180 nM, and therefore should not be considered on account of low binding affinity. The IC_50_ values of compounds **50**–**52**, meanwhile, were all in a promising range. It proved difficult to compare compounds **50**–**52**, however, because of the heterogeneity in FRET assay and eosinophil shape change assay results, in which compound **50** had values of 5 and 13 nM, compound **51** had values of 6 and 6 nM, and compound **52** had values of 0.8 and 10 nM, respectively. An interesting case was presented with compound **56**, for which the FRET assay IC_50_ was an acceptable 7 nM but the eosinophil shape change assay IC_50_ was 57 nM. None of these compounds have been further characterized.

In a further assessment of compound potency, a basophil chemotaxis assay was run in which the IC_50_ values were well correlated to those found in the FRET assay. Meanwhile, the similarly strong correlation between the FRET assay and hGPR44 binding assay with [^3^H]PGD_2_ confirmed the GPR44 binding potency. Eosinophil shape change assays of the promising compounds **45** and **47** confirmed the IC_50_ of the FRET assay, with only the slight change in compound **45** IC_50_ to 4 nM.

To establish the selectivity of the compounds, a cAMP time-resolved FRET (TR-FRET) assay with the lymphocyte NK-92 cells (DP_1_) and thromboxane B2 (TXB2), an inactive metabolite of thromboxane A2 (TXA2), the major ligand of TP, was carried out. Undisclosed representative compounds from the series were tested and found to have a selectivity of three orders in magnitude.

In the Caco-2 cell assay at pH 6.5, compounds **46**–**47** exhibited adequate permeability at 4.3 and 7.1 × 10^−6^ cm/s, whereas compound **49** had a slightly lower permeability at 2.1 × 10^−6^ cm/s. The increased permeability of these compounds indicates that adsorption occurs in the jejunum microvilli, due to this region’s slightly acidic pH. Although pharmacokinetic data was not further characterized, the Caco-2 cell assay results suggest that for compounds **46**–**47** and **49**, the permeability will not hamper adsorption or oral bioavailability. Interestingly, compound **47** exhibited a high plasma protein binding of 98% in an equilibrium dialysis experiment, but this was determined not to bar membrane permeability.

In addition, the compounds were found to bind at similar values to human and murine GPR44, indicating little concern if mouse models are to be used for in vivo studies. As such, an oxazolone-induced contact hypersensitivity (CHS) mouse model was then used to test compounds **45** and **47**, selected due to their permeability, the close correlation between the FRET and eosinophil shape change assays, and structure diversity and potential toxicity.

At an oral dose of 3 mg/kg twice daily, compounds **45** and **47** exhibited a close 34% and 30% reduction in swelling. In rats, compounds **45** and **47** exhibited similar optimal properties, with a clearance of 13 and 12 mL/min/kg, the half-life of 4.3 and 3 h, and steady-state volume of distribution of 1.7 and 1.6 L/kg, respectively. The AUC of compound **47** was higher at 5066 h·kg·ng/mL·mg relative to the 3568 h·kg·ng/mL·mg of compound **45**, which corresponded well to the increased C_max_ (772 ng/mL as opposed to 484 ng/mL) and bioavailability (37% as opposed to 28%) of compound **47**. For compound **47** specifically, the potential for drug-drug interaction was thought to be negligible, as evidenced by the CYP inhibition IC_50_, which ranged from 16 to 100 µM. In addition, the AMES test for identifying mutagens showed compound **32** had no effect, as well as no inhibition of human ERG at 33 µM compound **47**.

The promising compound **48** was also assessed in mice and showed lower clearance (9 mL/min/kg), a comparable half-life (4 h), a lower steady-state volume (1.12 L/kg), a higher AUC (7526 h·kg·ng/mL·mg), a higher C_max_ (1889 ng/mL), and a higher bioavailability (41%) as compared to compounds **45** and **47**. However, compound **48** was not further profiled.

On account of its more optimal profile, compound **47** was extensively characterized, starting from the determination of a minimum effective dose at 1 mg/kg for house dust mite (HDM)-induced allergic airway disease in mice. At an oral dose of 20 mg/kg daily, compound **47** inhibited airway inflammation and leukocyte counts, save for that of macrophages, in bronchoalveolar lavage. H&E staining confirmed the inhibitory effect of compound **47**, showing that it could decrease cell infiltration and epithelial and blood vessel hyperplasia and hypertrophy.

The efficacy of compound **47** was also evaluated in a sheep model of Ascaris-induced airway bronchoconstriction and hyper-responsiveness. Induced responses of both early and late phase allergic airway bronchoconstriction, as well as inflammation and hyper-responsiveness, responded to 3 mg/kg intravenous compound **47** twice daily, and apparently created a comparable effect at even 1 mg/kg intravenously. In addition, 3 mg/kg intravenously applied compound **47** attenuated the length of time of tracheal mucus velocity from 50% to 70% of baseline at 4 h [91]. These compounds have been used by Babu et al. in a machine-learning model to study the structure-activity relationship, which offers more insight on the observed behavior of these compounds. We will provide recommendations after reviewing Babu et al.’s publication in the next section.

#### 2.5.2. Mathematical Modeling

In 2016, Babu et al. at SRM University evaluated GPR44 antagonists using a series of mathematical models to assess the quantitative structure-activity relationship [92]. Specifically, these models included Comparative Molecular Field Analysis (CoMFA) to predict structure-activity relationship, Comparative Molecular Similarity Indices Analysis (CoMSIA) to correlate molecular properties with binding affinity, Topomer CoMFA to assist alignment issues with CoMFA, and Hologram Quantitative Structure Activity Relationship (HQSAR) for specialized molecular signatures. They used compounds disclosed by Kaila et al. to make up a training set to establish the model, and a test set to validate the model.

In addition, the activity structure fragments’ contribution to the molecule was determined, and an IC_50_ value was predicted to assess the validity of the model for some of Kaila et al. compounds, including compounds **36**–**40**, **42**–**43**, **45**–**48**, **50**–**56**, and **58** discussed above (Figure 7, Figure 8 and Figure 9). In all, the predicted IC_50_ matched well to the actual IC_50_. Table 3 lists the actual properties and calculated properties of these compounds.

The compound with the highest activity reported by Kaila et al. was compound **52**, with an IC_50_ of 0.8 nm. Thus, this compound was further modeled for its steric and electrostatic contours. Specifically, for compound **52**, the 5-position of the indole ring proved conducive to activity, particularly the electronegative atom, but the 7-position did not.

Indicated by the raised IC_50_, compounds **40** and **42** were structurally different from compound **52**, with replacements at the 5 and 7 regions of the indole core. In one of the fragments, the para benzyl ring and ortho diazine lent an increase in binding affinity.

The regional contribution to binding affinity was characterized for compound **52**. For instance, the 5-position fluorine and 4, 6, and 7-position hydrogen of the indole core, as well as the diazine ring, again were shown to contribute to compound **52′**s inhibitory qualities.

In compounds **36**–**40** and **42**–**44**, the meta benzyl ring also contributed to inhibitory qualities; however, when there was no co-expression of the pyridazine linker as in compound **42**, the meta benzyl ring lost this quality. In compound **47**, meanwhile, the diazine ring had high activity, whereas compound **42** exhibited activity at hydrogen at position 7 in the indole core.

The highly active compound **52** was further characterized for steric and electrostatic contours as part of CoMFA analysis, in which the ortho and meta-attached benzyl on diazine was seen to supplement inhibitory activity. For compound **37**, however, the methyl sulfur dioxide at the 5-position of the indole core was instead detrimental to activity. In addition, compounds **40** and **42** exhibited lower activity due to the replacement of the 5-position fluorine in the indole core, indicating the importance of the original fluorine.

Further characterization of compound **52** in CoMSIA contour maps confirmed previous results, as well as indicating that compounds **47**–**48** shared the beneficial para position of the benzyl group; this may have been due to a hydrophobic group at the para position. Likewise, an increase in affinity due to the 5-position of the indole ring may be attributed to a hydrophobic group at the position. In compounds **47**–**48** and **52**, the hydrophobic group was fluorine at the para position of the benzyl group. However, hydrophobicity at the 7-position of the indole core and meta position of the diazine ring resulted in decreased affinity of compounds **40** and **42**. The effects of an H-bond donor group were also important to consider, as the H-bond donor in the hydroxyl group of the carboxylic acid attached to the indole core facilitated improved binding. In contrast, an H-bond donor group at the meta and para position of the benzyl ring was detrimental to binding potency [92].

As Babu et al. reported, the modeling overall identified the presence of “(a) sterically bulky, electronegative, hydrophobic atom at para position of benzyl ring, (b) electronegative and hydrophobic atom at 5th position of indole ring could improve the activity of these compounds. The pyridazine linker and carboxylic acid are very important to make the compound more potent with higher activity” [92]. Based on these findings, the authors designed molecules based on our findings and found that the predicted activities of newly designed molecules were in the range of highly active molecules used in this study.

#### 2.5.3. Recommendations

The low IC_50_ values of compounds **45**–**49**, as well as compounds **50**–**52**, mark their potential for use as GPR44 PET tracers. As compound **47** has been extensively characterized and found to be efficacious, this compound may be particularly promising. The lack of extensive testing, though, does not preclude compounds **45**–**49** for further development.

In addition, several structural features, including electronegativity and hydrophobicity at the para position of the benzyl ring as well as the 5-position of the indole core were found to lead to improved inhibition. The presence of pyridazine linkers and carboxylic acids were also identified as lending significant qualities to the inhibitors. These findings of this modeling may well-inform the development of future GPR44 antagonists.

### 2.6. Almirall Analogues

#### 2.6.1. Almirall Analogues in 2013

**Figure 10:**
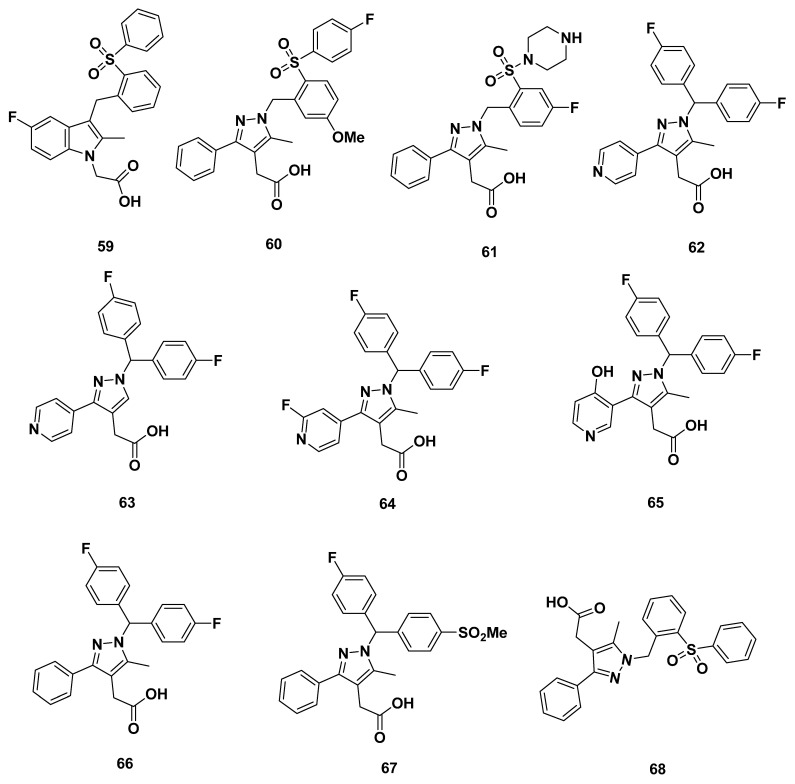
Molecular structures of compounds **59**–**68**.

Andrés et al. from Almirall synthesized a series of GPR44 antagonists on the basis of expanding the ring of the indole core in 2013 [93]. High throughput screening of 100 compounds by homogeneous cAMP assay yielded the initial pyrazole-4-acetic acid precursor substructure. The ortho-sulphonyl benzyl tail containing compound **59** (Figure 10) exhibited a low IC_50_ in both the eosinophil shape change assay from a reference that could not be located but which was referred to by Andrés et al., and GTPγS binding assay, which measured GPR44 signal transduction; the values were 5 and 16 nM, respectively.

Further changes, including separating the indole core to benzene ring connected with a pyrazole ring, resulted in compounds **60**–**61**. Compound **60** had a high GTPγS IC_50_ of 36 nM, indicating subpar binding affinity. The addition of a strong basic terminus group in compound **61** resulted in a much worse binding affinity where GTPγS IC_50_ = 5000 nM.

Based on the optimization of the substituents of the pyrazole core and dibenzyl derivative tail groups, compounds **62**–**67** were created. Unfortunately, the binding affinity of all the compounds was poor at GTPγS IC_50_ greater than 46 nM, with a median IC_50_ of 705 nM.

Other compounds in the series were profiled, including compound **62** with its IC_50_ of 46 nM, in which all showed <40% inhibition at 10 µM for DP_1_ and the TP. Dissociation kinetics were elucidated from membranes using the GTPγS assay, in which most compounds had rapid dissociation. The slowest dissociating compound was compound **62** with its half-life of 30 min. Although none of these compounds (save for **62**) contained the necessary fluorine group, the results may be representative of general compounds with an expanded indole core.

In all, compound **68** in the series was further profiled, but that compound did not contain the necessary fluorine group. The profile showed qualities characteristic of a lipophilic carboxylic acid, with adequate absorption, low clearance, and a long terminal half-life. However, overly high amounts of plasma protein binding were concerning. The compounds **60**–**61** may be presumed to have similar properties, but the later compounds (**62**–**67**), based off a polar pyridyl substitution, should not have concerns with decreased plasma protein binding and receptor ligand interactions [93].

#### 2.6.2. Recommendations

As such, compounds **59**–**60** may be considered on the basis of their IC_50_ values. Caution should be taken for compounds **60** on account of the plasma protein binding and subsequent detriments to receptor ligand interactions.

#### 2.6.3. Almirall Analogues in 2016

**Figure 11:**
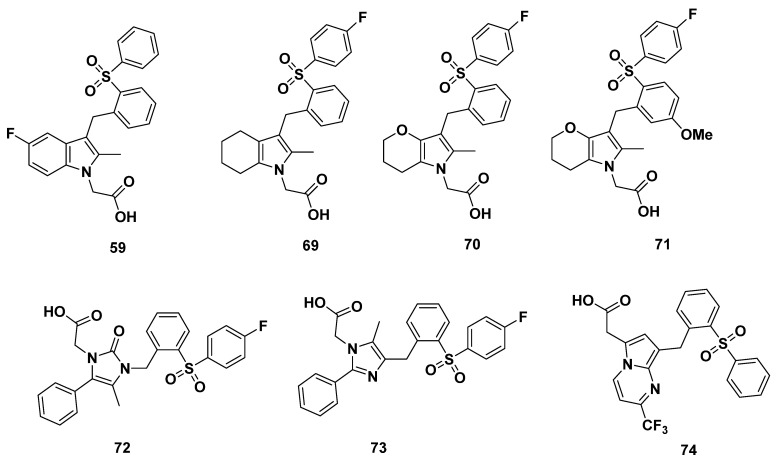
Molecular structures of compounds **59**, **69**–**74**.

Buil et al. of Almirall synthesized a series of GPR44 antagonists with differing cores (Figure 11) [94]. In 2016, they further described compound **59**, an indole GPR44 antagonist, with a moderate GTPγS IC_50_ of 14 nM and adequate dissociation half-life of 1.3 h.

Another compound, **69,** showed a high GTPγS IC_50_ of 104 nM. On the other hand, the pyrrole analogues **70**–**71** exhibited exactly the same GTPγS IC_50_ of 2 nM and dissociation half-life of 21 h. The low IC_50_ indicates high binding affinity, and the lack of differences between the methoxy-fluoro tail substitution of **71** and mono-fluoro substitution of **70** indicates a lack of their relevance to receptor binding. The long half-life indicates that the compounds may reside for a long time in vivo, perhaps due to the stability imparted by the extra-carbonyl group. In addition, the para-fluoro substituent in the tail may also impart a structure less prone to decomposition [94].

Changing the core to have the structure of imidazolone (compound **72**), imidazole (compound **73**) or pyrrolopyrimidine (compound **74**), compound **73** with an imidazole core appeared to be superior than the other two, with a GTPγS IC_50_ of 8 nM, while compound **74** had an elevated GTPγS IC_50_ of 47 nM, and that of compound **72** was as high as 10,000 nM.

#### 2.6.4. Recommendation

As the compounds **59**, **70**–**71,** and **73** all have moderate to good binding affinity to GPR44, they may all be considered. However, additional characterization would prove useful.

### 2.7. Actelion Analogues

**Figure 12:**
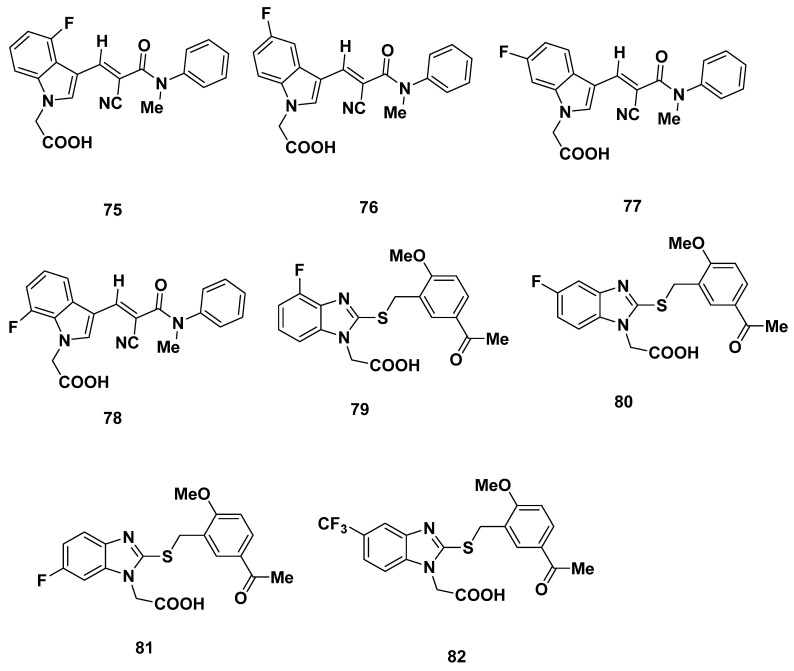
Molecular structures of compounds **75**–**82**.

In 2013, Valdenaire et al. from Actelion screened around 80,000 chemical compounds for their GPR44 binding affinity using high-throughput screening [95]. An assessment of properties led Valdenaire et al. to develop a series of compounds based on 1H-indole-3-carbaldehyde, all of which possessed a fluorine atom. Among them, compounds **75**–**78** (Figure 12) were assessed for inhibitory qualities through a Ca^2+^ assay in human embryonic kidney cells (HEK-293). While compounds **77**–**78** had IC_50_ values of 45 and 100 nM, respectively, compounds **75**–**76** were decidedly more promising with IC_50_ values of 10 nM [96].

Another series of compounds were designed to have a thiobenzimidazole core, including compounds **79**–**82** (Figure 12). Among these compounds, **79**–**80** and **82** each exhibited a desirably high GPR44 affinity with an IC_50_ of 5, 2 and 3 nM, respectively, while compound **81** showed a relatively higher value of 45 nM. Compound **80** was characterized more extensively, where it showed consistently high GPR44 affinity under different assay conditions, high selectivity over DP1 (with IC_50_ over 10,000 nM), good chemical and human plasma stability (e.g., 100% of the parent was recovered after incubation in human plasma for 4 h, and 95% was recovered after incubation in simulated gastric fluid (SGF) for 1 h), as well as good solubility in buffer solutions (399 ng/mL or higher). The intrinsic clearance (Cl_int_) in rat liver microsomes (RLM) was 60 µL/min/mg protein and was 20 in suspended hepatocytes (RHepa) µL/min/10^6^ cells and 13 µL/min/mg protein in human liver microsomes (HLM).

Compound **80** was a weak inhibitor of the cytochrome CYP450 isoform CYP2D6 and CYP2C9, with IC50 values higher than 50 µM in each case. Low inhibition of CYP3A4 was observed with an IC_50_ of 10 and 15 µM in mid and test conditions each. Compound **80** was given to Wistar rats at an oral dosage of 10 mg/kg and showed an acceptable 19% oral bioavailability [95].

#### Recommendations

Although the IC_50_ values of compounds **75**–**76** appear to be a reasonable basis for recommending their development into GPR44 PET tracers, further characterization of pharmacokinetic properties or in vivo assessments, for instance, would be of much use. On the other hand, represented by the thorough characterization of **80**, the series including **79**–**80** and **82** appear to have good properties that make them candidates to be developed as PET tracers.

## 3. Cyclopentenylindole-Based Potential GPR44 Analogues

Here, we describe a series of analogues sharing a common cyclopentenylindole group.

### 3.1. Actimis Analogues

**Figure 13:**
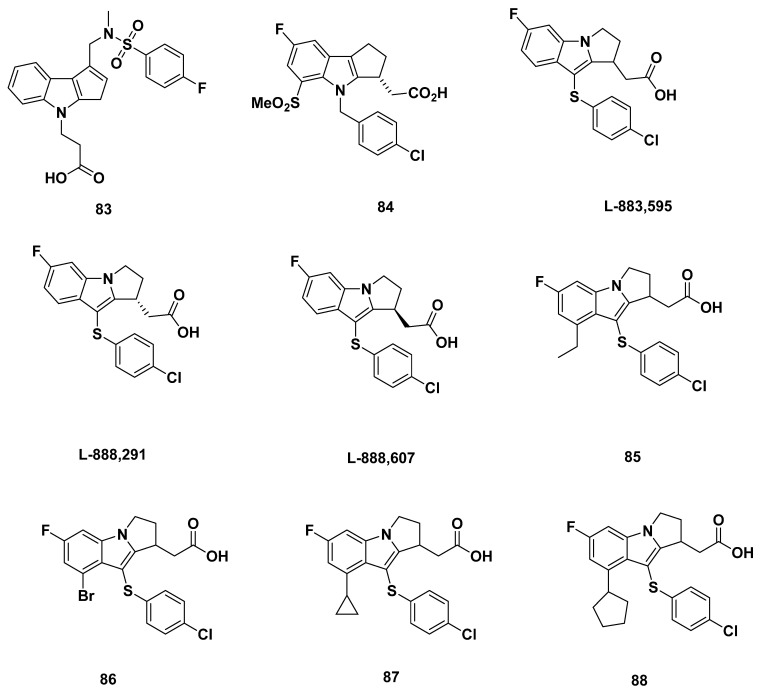
Molecular structures of compounds **83–88**, L-883,595, L-888,291, L-888,607.

In a 2005 review paper by Ly et al. from Actimis [97], compound **83** (Figure 13) was characterized. The compound description was based off a 2003 patent application WO 03/09742 from Shionogi in Japan. In place of the conventional acetic acid attached to the nitrogen atom of the indole group, compound **83** had an ethanoic acid. It also contained a p-fluorophenyl sulfonamide attached to the cyclopentenyl ring. The compound was reported to inhibit GPR44 at an IC_50_ value of 37 nM.

#### Recommendations

Compound **83** does not have a particularly promising IC_50_ value, so we recommend further characterization of its properties.

### 3.2. Merck Analogue

#### 3.2.1. Merck Analogue in 2005

In 2005, Campos et al. from Merck characterized the asymmetric synthesis of a reported PGD_2_ receptor antagonist **84** (Figure 13), but no further details about binding affinity were provided [98].

#### 3.2.2. Recommendations

Due to the paucity of information at this time, we are unable to make a recommendation about this compound.

#### 3.2.3. L-888,607, L-883,595, and L-888,291

In 2005, Gervais et al. from Merck developed L-888,607 (Figure 13), reportedly the first synthetic, potent, and selective GPR44 agonist [69].

At first, the racemic compound L-883,595 (Figure 13) was identified as a base for modification through screening. Although the affinity to GPR44 was optimal at a K_i_ of 4 nM, L-883,595 was shown to have potential off-target binding to DP_1_ at a K_i_ of 211 nM, with both values measured through binding assays.

Separating the enantiomers of L-883,595 resulted in the L-888,291 (Figure 13) and L-888,607, which were the R and S enantiomers, respectively. **L-888,291** was not promising, as it had an approximately equal binding affinity for GPR44 and DP_1_ (48 and 40 nM, respectively).

L-888,607, meanwhile, was promising with a high binding affinity for GPR44 at a K_i_ of 0.8 nM, and a less concerning binding affinity for DP_1_ (2331 nM) that was over 1000 times of that for GPR44.

A series of L-883,595 analogues were also characterized, in which substitution at the 6-position of the indole ring was explored. However, all did not exhibit as promising an affinity to GPR44 as L-888,607, although compound **85**, produced through substitution by an alkyl group, showed the lowest K_i_ at 12 nM. Other compounds included compound **86**, with a K_i_ of 21 nM, compound **87**, with a Ki of 20 nM, and compound **88**, with a Ki of 94 nM.

Further equilibrium binding assays characterized promising properties of L-888,607, with a binding affinity (K_i_) 360-fold lower for the TP receptor and more than 1000-fold lower for six other prostanoid receptors (EP_1_–EP_4_, FP, and IP). Additionally, potential off-target binding to chemokine receptors (CCR1, CCR2, CCR4, CCR5, CXCR1, CXCR2, and CXCR3), anaphylatoxin receptors (C2aR and C5aR), and the cyclooxygenases-1 and -2 was shown to be minimal, as no significant binding was found at 10 µM L-888,607.

L-888,607 was additionally confirmed as a strong inhibitor through measurements of cAMP inhibition (EC_50_ = 0.5 nM), eosinophil shape change induction, and eosinophil chemotaxis (at an optimal concentration of 100 nM).

The pharmacokinetic profile was elucidated following intravenous or oral administration to mice. The results showed no causes for concern, with a maximum concentration of 36.1 M, half-life of 2.9 h, trough level at 8 h of 3.5 M, and AUC_0–8h_ of 87.1 M after intravenous administration of 5 mg/kg L-888,607. The oral administration of 20 mg/kg L-888,607 showed similar values, with a maximum concentration at 31.6 M, half-life of 4 h, trough level at 8 h of 15.4 M, and AUC_0–8h_ of 166 M. The bioavailability was sufficient at 48%.

#### 3.2.4. Recommendations

As a result of the high binding affinity and specificity to GPR44, as well as the optimal in vivo pharmacokinetic properties, L-888,607 can be considered for the development of a GPR44 PET tracer.

#### 3.2.5. MK-0524

**Figure 14:**
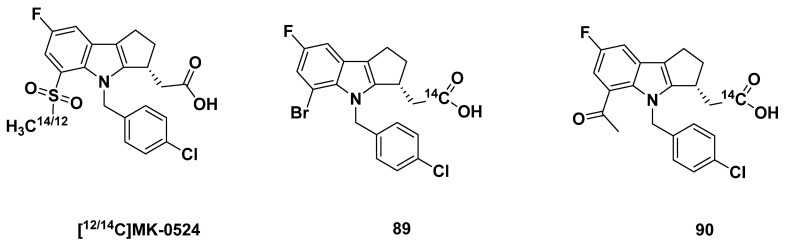
Molecular structures of compounds MK-0524, **89**–**90**.

MK-0524, [(3R)-4-(4-chlorobenzyl)-7-fluoro-5-(methylsulfonyl)-1,2,3,4-tetrahydrocyclopenta[b]indol-3-yl] acetic acid, also referred to as laropiprant commercially, was developed by Sturino et al. from Merck in 2007 (Figure 14) [82]. It was initially targeted for the treatment of niacin-induced flushing and was also considered a promising compound to alleviate allergic disorders. MK-0524 was a selective DP antagonist, demonstrating potent inhibition of PGD_2_-induced cAMP production with a K_i_ of 0.57 nM, an IC_50_ of 0.09 nM in washed platelets, and an IC_50_ of 4.0 nM in platelet-rich plasma [82]. In contrast, MK-0524 exhibited much lower selectivity for TP, as shown by its K_i_ of 2.95 nM and IC_50_ of 770 nM in platelet-rich plasma [82].

Due to its desirable selectivity for DP_1_ and therapeutic potential, MK-0524 has been characterized extensively. A study intravenously administered [^14^C]MK-0524 in rats, dogs, and monkeys and evaluated its metabolism, pharmacokinetics, and excretion [99]. MK-0524 was metabolized primarily through acyl glucuronidation into the major metabolite, acyl glucuronide, and subsequently excreted via the bile ducts in both rats and dogs. The minor metabolites, monohydroxylated epimers and the keto-metabolite, occurred in trace amounts [100]. Following intravenous dosing at 1 and 5 mg/kg, rats exhibited a low plasma clearance of 2 mL/min/kg, a small volume of distribution of 0.8 L/kg, and a moderately long half-life of 7.5 [99]. The plasma concentrations of acyl glucuronide remained low compared to the parent compound. In dogs receiving 1 and 5 mg/kg intravenous doses, the mean values reflected a similar trend at 6 mL/min/kg, 5 L/kg, and 13 h. Unlike rats, the plasma concentrations of acyl glucuronide in dogs were substantial with AUC _(0–∞)_ and C_max_ values ranging from 8 to 18% for MK-0524. The values varied in monkeys after 3 mg/kg intravenous administration, in which the plasma clearance was 8 mL/min/kg, the volume of distribution was low at 1 L/kg, and the half-life was 3 h. Acyl glucuronide plasma concentrations were higher in monkeys than in rats and dogs, with an AUC _(0–∞)_ value of 27%. Thus, the ratio of acyl glucuronidation to the parent compound was found to be species-specific.

Another study showed that MK-0524 underwent qualitatively similar metabolism in humans [100]. Extensive glucuronidation in hepatocytes and liver microsomes was found using human liver microsomes, human intestinal microsomes, and recombinant human uridine diphosphate glucuronosyltransferases. In a clinical study, the metabolism and excretion of [^14^C]MK-0524 were evaluated in six healthy human participants using 40 mg of oral administration [101]. The results confirmed that the main metabolite was acyl glucuronic acid conjugate, which was by excretion into bile and then feces.

#### 3.2.6. Recommendations

A significant disadvantage of MK-0524 is its off-target DP_1_ agonist activity. Therefore, MK-0524 is not a suitable molecule for the development of a GPR44 tracer.

#### 3.2.7. Merck Analogues in 2007

In 2007, Sturino et al. from Merck reported two additional analogues that exhibited improved DP_1_ selectivity [82]. The bromide compound **89** (Figure 14) was measured for DP_1_ binding affinity, and showed a K_i_ 1.5 nM. The methylketone compound **90** (Figure 14), a transformed product of compound **89**, had a similarly strong binding affinity at a K_i_ of 1.1 nM. The IC_50_ values for DP in washed platelets and platelet-rich plasma were similar for both compounds **89** (0.26 nM and 12 nM, respectively) and **90** (0.29 nM and 15 nM, respectively), although the platelet-rich plasma K_i_ values were interestingly much higher than the other assays.

The ligand-receptor interactions of compound **89′**s binding to DP_1_ were elucidated by Mu et al. of Sanofi Aventis in 2011 [83].

Similarly, the IC_50_ values for binding to TP in platelet-rich plasma were higher at 80 and 1400 nM, respectively. The concern of the selectivity of both compounds was further confirmed by K_i_ values, as compounds **89**–**90** exhibited a binding affinity for TP of 14 and 0.84 nM, respectively.

As measured in rat hepatocytes, compounds **89**–**90** were shown to be largely metabolized, with only 42% and 35% remaining, respectively, following 2 h incubation. In pharmacodynamic characterizations of the compounds in rats, compounds **89**–**90** continued to show optimal properties, including adequate half-lives at 12 and 4 h, respectively, moderate bioavailability at 41% and 52%, respectively, and low plasma clearance of 1.4 and 1.9 mL/min, respectively. The C_max_ (10 and 21 10 mg/kg, respectively) concentration 6 h post-dose (3.7 and 7.5 10 mg/kg, respectively), and volume of distribution (0.4 and 0.5, respectively) values also were of little cause for concern.

In the metabolic route, intravenous cannulation surveying found that compounds **89**–**90** did not accumulate in the bile fluid, as C_max_ was 29 and 91 µM, respectively. The t_max_ of the compounds reflected the maximum concentration at values of 1–1.5 h and 2.5–3 h, respectively. Hydroxylation and glucuronidation were cited as the main routes of metabolism.

In further investigation of the profile of compounds **89**–**90**, the compounds were radiolabeled with [^14^C] and studied in rat and human microsomes and hepatocytes. The covalent binding of compound **89** was equivalent at 33 pmol equiv/mg of protein per h for both human microsomes and hepatocytes, but varied from 460 to 74 pmol equiv/mg·h, respectively, in rat microsomes and hepatocytes. The variation for compound **90** was more severe with rat microsomes (290 pmol equiv/mg·h), rat hepatocytes (45 pmol equiv/mg·h), human microsomes (46 pmol equiv/mg·h), and human hepatocytes (16 pmol equiv/mg·h) all differing. The microsomal system often afforded values higher than the hepatocyte system, which may be due to the action of reactive intermediates. The high binding in at least the rat system was promising, but the covalent binding was low in the human system. These disparate results may indicate species-specific antagonism, potentially limiting the in vivo testing of compounds **89**–**90**.

#### 3.2.8. Recommendations

Due to the uncertainty of the binding affinity to GPR44, strong off-target binding to DP_1_, and potential off-target binding to TP, compounds **89**–**90** are not recommended.

#### 3.2.9. Merck Analogues in 2008

**Figure 15:**
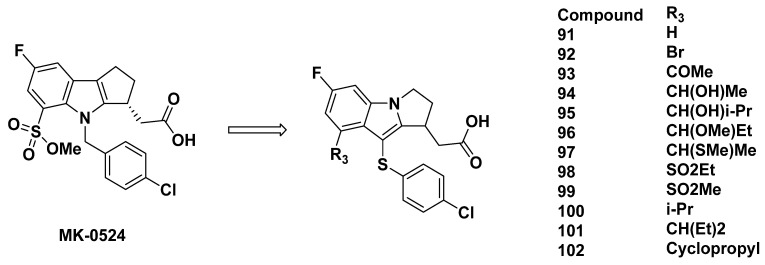
Molecular structures of compounds **91**–**102**.

Another series of compounds based on MK-0524 was developed by Beaulieu et al. from Merck in 2008 [102]. In these compounds, the nitrogen atom and 3-position carbon on the indole of MK-0524 were exchanged (Figure 15).

The compounds **91**–**102** (Figure 15) resulted from substitutions at the 4-position carbon on the indole group. Among the various substituents, compounds **96** and **99** showed good binding affinity to DP_1_ with K_i_ values of less than 2 nM and IC_50_ values in platelet-rich plasma of 6.7 and 10.4 nM, respectively. The selectivity was also strong, as the compounds were 170-fold more selective for DP_1_ than TP. The remaining compounds did not exhibit either high enough binding affinity or selectivity for DP_1_.

**Figure 16:**
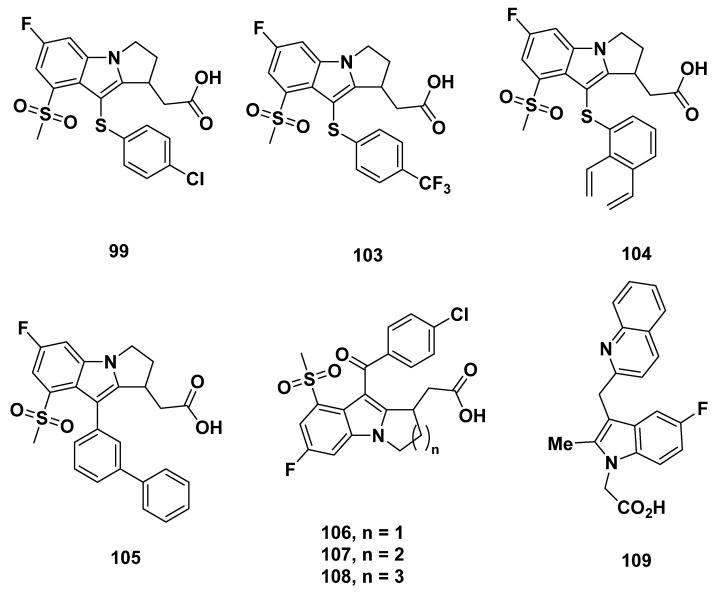
Molecular structures of compounds **99**, and **103**–**109**.

Through substitution of the 3-position carbon of the indole group, compounds **103**–**105** were developed (Figure 16). All of the compounds were strong DP_1_ antagonists, with a K_i_ of less than 2 nM and IC_50_ at most 40 nM. The selectivity for DP_1_ over TP was also over 150-fold.

Further development led to compounds **106**–**108**, in which the number of carbons on the indole-fused ring differs. The binding affinity, represented by K_i_ and IC_50_ values, appeared to decrease as ring size increased. The K_i_ value was the lowest for compound **108** at 1.4 nM and IC_50_ of 5.2 nM, although the selectivity may be a cause for concern at a TP K_i_ of 675 nM. Whereas compound **107** exhibited similar properties to compound **108**, compound **106** had a much greater selectivity with a TP K_i_ of 6972 nM, but lowered binding affinity at a K_i_ for DP_1_ of 6.9 nM.

#### 3.2.10. Recommendations

As a consequence of the unidentified GPR44 binding affinity and potential off-target binding to DP_1_, these compounds may not serve as optimal GPR44 PET tracers.

### 3.3. Astellas Analogues in 2008

Compound **109** (Figure 16) was briefly described as an indole acetic acid in a 2012 paper by Ito et al. from Astellas, and cited in a 2005 patent by Middlemiss et al. [103].

#### Recommendations

We are unable to make a recommendation due to the lack of information about this compound.

## 4. Potential Fluorination Methods

According to the strategies discussed above, Eriksson, O. et al. have developed ^11^C-MK-7246 [104,105] and ^18^F-MK-7246 [106] from MK-7246, and our team has developed ^18^F-TM30089 from TM30089 and two other ^18^F-labeled GPR44 PET tracers [107]. These potential ^18^F-labeled PET tracers displayed high specific binding to GPR44 in murine pig and monkey. It validated the upside of such a strategy, i.e., the prominent GPR44 ligands have been thoroughly characterized (affinity, lipophilicity, protein binding, etc.), which makes it a highly efficient process to screen the most promising GPR44 PET tracers.

**Figure 17:**
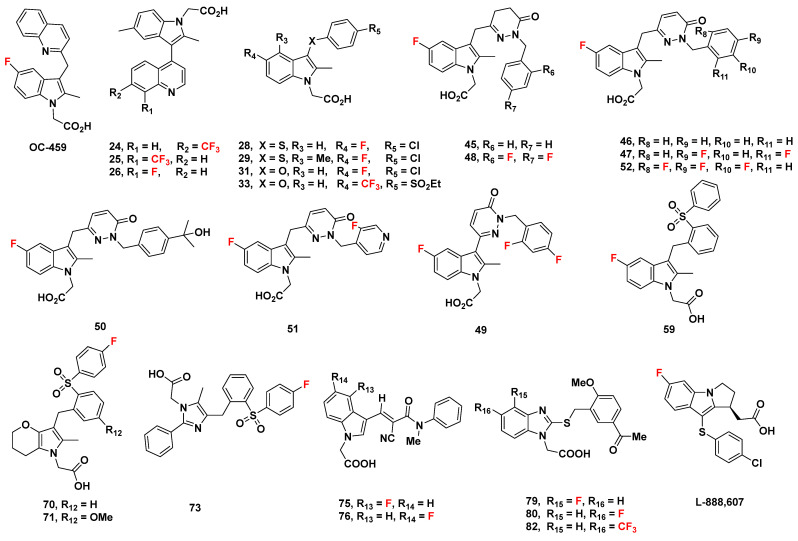
All potential “cold” ^18^F-labelled GPR44 candidates. F is shown in red color.

For the radiolabeling strategies for developing ^18^F-labeled GPR44 ligands from “cold” GPR44 candidates (Figure 17), there are many publications about methodologies for ^18^F-labeling [108,109,110]. Direct and indirect radiolabeling methods may be utilized to incorporate fluorine-18.

### 4.1. Concerted Nucleophilic Aromatic Substitution (CS_N_Ar) with ^18^F^-^

**Figure 18:**
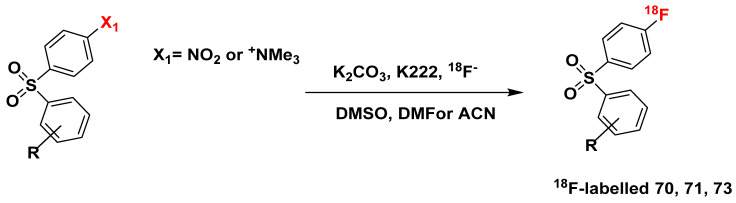
Potential labeling compounds **70**, **71**, and **73** by S_N_Ar. The labeling position is shown in red color.

Orit Jacobson (2015) stated that “^18^F nucleophilic aromatic substitution (S_N_Ar) requires sufficient activation of the phenyl ring, which can be achieved by electron withdrawing group(s) (such as −NO_2_, −CN, −CF_3_, carbonyl, or sulfur dioxide groups) in the *ortho* or *para* position to the leaving group” [110]. The most common and efficient leaving groups for no-carrier-added nucleophilic aromatic substitutions are trimethylammonium salt and the nitro group [109]. This ^18^F-labeling method is potentially suitable for compounds such as compounds **70**, **71**, and **73** (Figure 18). Lower temperatures of fluorination correspond to the precursors with a trimethylammonium group compared with nitro group-containing precursors.

Traditional two-step S_N_Ar (nucleophilic aromatic substitution) requires the presence of an electron-withdrawing group located ortho or para to the leaving group, a constraint that severely limits its applicability. In order to enable the rate-determining step of S_N_Ar (addition of the ^18^F nuclide to form the Meisenheimer complex), the ring must be pi-deficient, as only pi-deficient arenes can adequately stabilize the anionic Meisenheimer complex [111].

**Figure 19:**
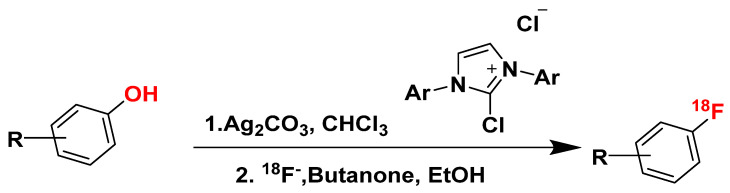
Potential labeling method by CS_N_Ar for almost all promising GPR44 candidates except compounds **24**, **25**, **33**, and **82**. The labeling position is shown in red color.

However, a concerted nucleophilic aromatic substitution reaction (CS_N_Ar) has been presented by Constanze N. Neumann et al., in which substitution is not restricted to electron-deficient arenes, as it does not proceed via the formation of a Meisenheimer intermediate (Figure 19) [112]. They show that the deoxyfluorination of phenols with PhenoFluor favors CS_N_Ar over conventional stepwise displacement, demonstrating that it is a viable alternative to two-step S_N_Ar. By combining the addition (nucleophilic attack) and elimination steps into a single, concerted step in CS_N_Ar, there is significantly less build-up of negative charge, lowering the activation energy barrier and removing the need for electron-withdrawing groups to stabilize the anionic intermediate.

One potential limitation may be present in the elution procedure outlined by Constanze N. Neumann et al., which, despite eliminating the need for the azeotropic drying of ^18^F-fluoride, cannot be used with substrates containing carboxylic acids, as carboxylic acid prevents the formation of key uronium intermediate [112]. There are certainly different ways to modify the procedure to accommodate substrates with carboxylic acid, but further experimental verification would be necessary to ensure viability. Pre-treating the carboxylate group of precursors with a cation [K^+^c2.2.2]_2_C_2_O_4_ to make a cationic chelate [K^+^c2.2.2]_2_CO_3_ could potentially improve this drawback.

CS_N_Ar is potentially suitable for labeling all 26 promising GPR44 candidates. However, while it can be a powerful method for the synthesis of radiolabeled compounds, potentially offering improved yields compared to traditional methods, each specific reaction still requires substantial experimental validation to precisely understand its efficacy.

### 4.2. Other Traditional Approaches

**Figure 20:**
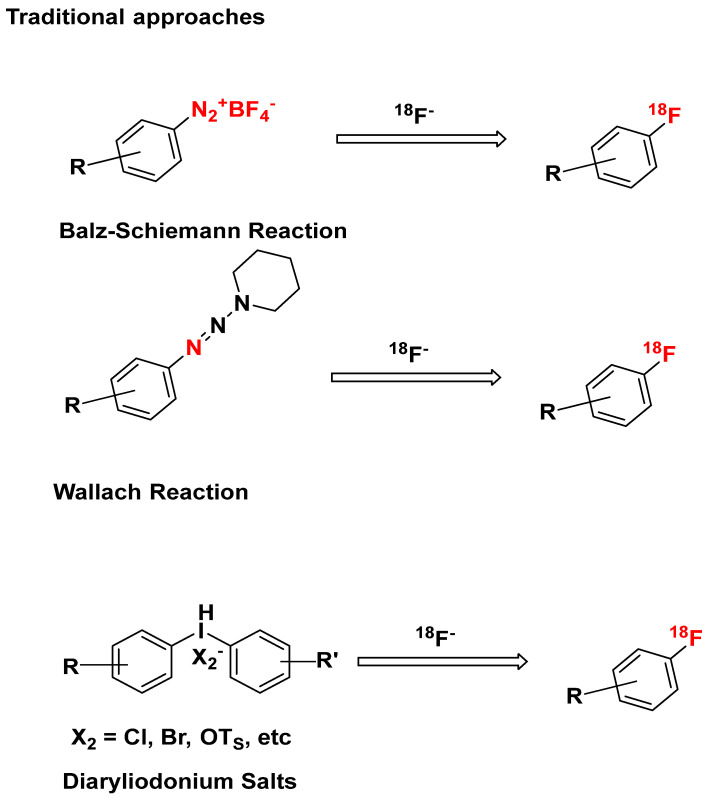
Potential labeling methods for labeling almost all promising GPR44 candidates, excluding compounds **24**, **25**, **33**, and **82**. The labeling position is shown in red color.

Labeling aromatic rings with ^18^F^−^ in the absence of S_N_Ar-activating groups and in a regiospecific manner could be accomplished (Figure 20) using the Balz−Schiemann reaction [113], Wallach Reaction [114], and Diaryliodonium Salts method [115,116].

The Balz-Schiemann reaction, used to convert aromatic amines into aromatic fluorides, involves the synthesis of a dry aryldiazonium tetrafluoroborate from an aromatic amine, followed by thermal decomposition into the desired aromatic fluoride, boron trifluoride, and nitrogen. The significance of this method arises from its wide applicability in fluorinating both electron-excessive and electron-deficient aryl amines.

However, it is limited by several factors: (1) substrate scope, restricted to primary aromatic amines, (2) low specific activity caused by competing incorporation of non-radioactive fluoride from monolabeled BF_4_^−^, (3) a maximum theoretical RCY of 25% due to isotopic exchange, (4) byproducts, as a significant amount of potentially toxic waste may be produced, (5) reaction conditions, as stringent anhydrous conditions and high temperatures are required [117].

These limitations have been at least partially addressed by modifications (e.g., photoredox catalysis, hypervalent iodine (III) catalysis, continuous flow, specialized low-polarity solvents, in situ diazotization with tert-butyl nitrite or [NO] [BF_4_], etc.) [118].

Because the Balz-Schiemann reaction is applicable only to primary aromatic amines, it may not be suitable for compounds **24**, **25**, **33**, and **82**. Additionally, because diazotiza-tion typically requires acidic conditions, it may not be suitable for compounds containing functional groups susceptible to hydrolysis (e.g., esters and amides). Strongly deactivating groups on the aromatic ring (e.g., –NO_2_, –CF_3_, –C(=O) R, –C(=O) H, –SO_3_H, etc.) may prevent formation of the diazonium ion. This reaction may be suitable for compounds OC-459, L-888,607, **26**, **28**, **29**, **31**, **59**, **79**, and **80**.

Another potential fluorination method, the Wallach reaction, involves the thermal decomposition of an aryl triazine to an aryldiazonium salt under acidic conditions. The greater stability of triazine compared to diazonium salts gives the Wallach reaction a significant advantage over the Balz-Schiemann reaction [118]. Additionally, triazene-masked diazonium ions can be utilized not only in solution-based reaction processes but also on solid substrates, making them compatible with different combinatorial techniques. However, the two-step elimination-addition process (through a cationic intermediate, which can react with any existing nucleophile) of the Wallach reaction could result in the production of multiple byproducts and subsequently a reduced RCY. More detailed information (for example, via further testing) regarding its functional group compatibility is necessary to determine whether or not it is suitable for fluorinating each GPR44 ligand [110].

Diaryliodonium salts, a category of hypervalent iodine (III) reagents possessing some covalent properties (demonstrated by X-ray structures), are used as versatile reagents in the synthesis of aryl fluorides. Their relative electrophilicity (compared to halide anion salts) and good solubility in most organic solvents makes them promising for use in nucleophilic aromatic fluorination [119].

A Cu-catalyzed approach using KF as a source of fluorine (explored by Naoko Ichiishi et. al.) possesses wide substrate applicability, high selectivity, and high functional group tolerance. This addresses several limitations of fluorination using diaryliodonium salts: high temperatures, aryl exchange limiting RCY, and production of unwanted regioisomers. (^t^BuCN)_2_Cu(OTf), Cu^I^(OTf)·benzene, Cu^II^(OTf)_2_ were all shown to be effective catalysts for the fluorination of compounds with the general structure [Mes–I–Ar]BF_4_ (with Ar representing an electron-rich aromatic ring), demonstrating considerable potential for further exploration with other traditionally challenging precursors. Cu catalysis also significantly enhanced selectivity and yield in substrates bearing electron-withdrawing groups as well [120].

The regioselectivity of electrophilic fluorination using ^18^F-F_2_ can be improved using aryltrimethyltin as a precursor, producing fewer byproducts and a higher RCY [110].

### 4.3. Novel ^18^F-Labeling Strategies

Wei Chen et al. has developed a method for the fluorination of aromatic C-H bonds using a [^18^F] F^-^ salt under blue light illumination, a strategy demonstrated to be effective in tracer studies conducted on mice. This method uses single-electron photooxidants to catalytically produce cationic arene radicals from the original aromatic substrates, serving as reactive intermediates that can more readily undergo substitution reactions via nucleophilic attack (thus enabling C-^18^F bond conversion) [121].

A later report demonstrates that polarity-reversed photoredox-catalyzed deoxyfluorination, proceeding via cation radical accelerated S_N_Ar, allows for the fluorination of electron-rich aromatic compounds at mild temperatures (mitigating the need for electron-withdrawing groups). Their approach targets the C-O bond in phenol derivatives, establishing a method for unsymmetrical diaryl ethers that relies on the preferential oxidation of the more electron-rich aromatic ring. Utilizing an acridinium-based photooxidant, [^18^F] TBAF, and excess phase-transfer reagent TBAHCO_3_ (minimizing Hofmann elimination of [^18^F] TBAF) under 450 nm LEDs, they obtained excellent RCYs while minimizing byproduct formation [122].

Ultimately, the feasibility of such reactions would highly depend on the specific substrates and reaction conditions. Thus, while theoretically possible, whether a specific photoredox-catalyzed S_N_Ar reaction could occur would need to be confirmed experimentally.

### 4.4. [^18^F] Trifluoromethylation (Compounds ***24***, ***25***, ***33***, and ***82***)

The earliest ^18^F-trifluoromethylation methods rely on halex exchange, a type of S_N_Ar involving the replacement of an aromatic halogen with a different nucleophilic halide. However, such techniques are limited in substrate scope (applicable only to electron-deficient arenes), radiochemical yield, and specific activity [123]. Furthermore, their usage of aryl difluoromethylene precursors with halide-based leaving groups necessitates stringent conditions to enable the rate-determining elimination step.

An alternative method, [^18^F] fluorodecarboxylation proceeding via the Ag(I)-catalyzed electrophilic addition of [^18^F] F^-^ from [^18^F] Selectfluor bis(triflate), has been previously used to synthesize ^18^F-trifluoromethylarenes. While it is applicable to a broader range of substrates and possesses wider functional group tolerance than nucleophilic halex exchange, its efficacy is limited by its operational complexity [123,124].

A more recent fluorination method developed by Gouverneur et al. in 2013 involves the Cu(I)-catalyzed single-operation installation of [^18^F]CF_3_ into a (hetero)aryl iodide precursor, directly forming [^18^F]CF_3_ (hetero)arenes and negating the requirement for an arylCF_2_ precursor possessing a distinct leaving group favorable for [^18^F] F^-^ substitution. In this approach, the (hetero)aryl-CF_2_-^18^F linkage is directly constructed through two main steps: (1) halide-induced decarboxylation of methyl chlorodifluoroacetate in the presence of [^18^F] F^-^, producing a trifluoromethide intermediate that can be captured by copper iodide to form [^18^F] CuCF_3_, (2) cross-coupling of [^18^F] CuCF_3_ with the (hetero)aryl iodide precursor, producing the desired [^18^F] trifluoromethyl (hetero)arene [124]. Riss et al. presents an alternative method for forming [^18^F] CuCF_3_ via a similar stepwise process that uses CHF_2_I in place of methyl chlorodifluoroacetate [125]. This approach offers several significant advantages, namely wide functional group tolerance, high RCY and regioselectivity, and amenability to a variety of bioactive molecules, which is especially relevant when developing potential GPR44 PET tracers. However, a limitation of this method is its incompatibility with unprotected amines, alcohols, and carboxylic acids. This could partially be resolved by protecting amine and carboxylate groups as carbamates and esters, respectively, then removing these protecting groups once the reaction is complete [126].

Cu(I)-catalyzed trifluoromethylation of heteroaryl iodides appears to be promising for the labeling of compounds **24**, **25**, **44**, and **82**, though experimental validation is necessary to ensure that this method is applicable to each specific target molecule.

## 5. Conclusions

In addition to ramatroban analogues evaluated previously, there are a number of indole-based and cyclopentenylindole-based compounds with fluorine atoms that demonstrated high affinity and selectivity to GPR44. These compounds have the potential to be developed into GPR44 ^18^F-labeled PET tracers for in vivo imaging of inflammation-related research, which can provide important insights into inflammation-cancer dynamics.

## Figures and Tables

**Table 1:** Activity profiles of compounds **1**–**6** [63,64].

Compd.	Binding *K*_i_ (µM)							IC_50_ (µM) ^b^
	mEP1	mEP2	mEP3	mEP4	hIP	mDP	hDP	hDP
**1**	>10	2.1	1.7	>10	0.26	0.023	0.0053	0.00081
**2**	0.27	0.24	1.4	1.9	0.026	0.0024	0.046	0.15
**3**	1.2	0.73	2.0	1.6	NT ^a^	0.019	0.20	0.37
**4**	0.20	0.71	2.8	3.5	0.29	0.0093	0.13	0.47
**5**	>10	2.1	1.7	>10	NT ^a^	0.023	0.0053	0.00081
**6**	>10	2.8	>10	>10	NT ^a^	0.11	NT ^a^	NT ^a^

^a^ NT: not tested.; ^b^ IC_50_ (µM): hDP receptor antagonist activity.; m/h: mouse/human.

**Table 2:** Effect of substitution of the N-benzoyl moiety on the activity profiles of compounds **8**–**16** [64].

Comp.		X	Y	Binding *K*_i_ (nM)	IC_50_^a^ (nM)	Binding *K*_i_ (nM)
				mDP		hIP
**8**	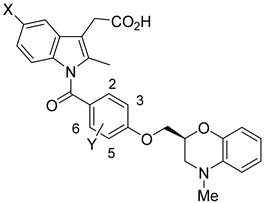	H	2-F	3.8	2.5	110
**9**		H	3-F	240	NT ^b^	210
**10**		F	2-Me	2.7	3.2	130
**11**		F	2-Cl	5.2	1.6	200
**12**		F	2-F	3.8	0.7	320
**13**		F	3-Me	59	42	160
**14**		F	3-OMe	NT ^b^	68	1500
**15**		F	2,3-Me	NT ^b^	32	230
**16**		F	2,5-Me	14	2.8	93

^a^ IC_50_ (nM): mDP receptor antagonist activity.; ^b^ NT: not tested.

**Table 3:** Actual and predicted pIC50 of compounds **36**, **37**–**40**, **42**–**43**, **45**–**48**, **50**–**56**, and **58** [90].

Compound	Actual pIC_50_	Predicted pIC_50_	Residual
**36** *	7.699	7.892	−0.193
**37** *	7.638	7.776	−0.138
**38**	7.046	6.902	0.144
**39**	7.119	6.990	0.129
**40** *	6.611	6.190	0.421
**42**	6.030	6.149	−0.119
**43** *	7.959	8.317	−0.358
**45**	8.824	8.971	−0.147
**46**	8.046	8.083	−0.037
**47**	8.523	8.453	0.070
**48** *	8.398	8.266	0.132
**50**	8.301	9.168	−0.867
**51**	8.222	8.177	0.045
**52**	9.097	9.120	−0.023
**53**	7.699	7.807	−0.108
**54**	7.921	7.889	0.032
**55** *	7.796	8.134	−0.338
**56**	8.155	8.109	0.046
**58**	7.553	7.663	−0.110

* Test set compounds for Topomer CoMFA.

## Data Availability

Data sharing is not applicable.
